# Phage-encoded sRNA counteracts xenogeneic silencing in pathogenic *E. coli*

**DOI:** 10.1371/journal.ppat.1014486

**Published:** 2026-08-18

**Authors:** Pranita Poudyal, Brandon Sy, Daniel G. Mediati, Michael Payne, Vibhuti Nandel, Dougall Norris, Sean McAteer, Asim Ullah, Serena Li, Lawrence Menz, Saleh Alquethamy, Jacob Scadden, Pietro Ridone, Shafagh Waters, Timothy J. Dallman, Mathew Baker, Ruiting Lan, David Gally, Jai J. Tree

**Affiliations:** 1 School of Biotechnology and Biomolecular Sciences, UNSW, Sydney, Australia; 2 Australian Institute for Microbiology and Infection, University of Technology Sydney, Ultimo, Australia; 3 The Roslin Institute, Division of Bacteriology, University of Edinburgh, Edinburgh, United Kingdom; 4 School of Biomedical Sciences, Faculty of Medicine and Health, UNSW Sydney, Sydney, New South Wales, Australia; 5 Public Health Laboratory Innovation Platforms, WHO Hub for Pandemic and Epidemic Intelligence, WHO Health Emergency Preparedness and Response Programme, World Health Organization, Berlin, Germany; Children's Hospital Boston, UNITED STATES OF AMERICA

## Abstract

Horizontal gene transfer introduces foreign DNA that can disrupt cellular processes and is therefore subject to xenogeneic silencing by nucleoid-associated proteins such as H-NS and Hha. In Enterohaemorrhagic *Escherichia coli* (EHEC), prophages make up a large fraction of the accessory genome and encode many virulence factors, yet their expression must overcome this silencing. We identify a prophage-encoded small RNA (sRNA), HnrS, that functions as an anti-silencing factor by targeting the H-NS paralogue Hha. HnrS is a short (66-nt) sRNA that is enriched in the locus of enterocyte effacement (LEE⁺) *E. coli* strains and present in up to nine copies in EHEC and Enteropathogenic *Escherichia coli* (EPEC) genomes. HnrS base-pairs with the *hha* ribosome-binding site to inhibit translation, thereby modulating Hha–H-NS repression of virulence loci including the LEE type III secretion system. Loss of HnrS alters motility, T3SS expression, and a subset of Hha-regulated genes. These findings reveal an RNA-based counter-silencing strategy encoded by prophage to relieve xenogenic silencing.

## Author summary

Horizontal gene transfer allows bacteria to acquire new traits, including many of the genes that make pathogens harmful. However, newly acquired DNA can also disrupt normal cellular functions, so bacteria have evolved systems that silence foreign genes until they can be safely integrated into existing regulatory networks. Disease-causing strains of *Escherichia coli* contain large amounts of foreign DNA carried by bacteriophages (viruses that infect bacteria), including many genes required for infection. How these genes escape silencing is incompletely understood. In this study, we identified a small RNA called HnrS that is encoded by bacteriophages integrated into the chromosome of pathogenic *E. coli*. We found that HnrS blocks production of Hha, a protein that helps silence foreign DNA. By reducing Hha levels, HnrS relieves repression of selected virulence genes, including those involved in host attachment. HnrS also influences bacterial motility and the expression of genes carried on prophages. Unlike previously described phage proteins that broadly interfere with bacterial silencing systems, HnrS is a small RNA that inhibits part of the silencing system that is specialised for virulence genes. Our findings reveal a new strategy used by bacteriophages to influence bacterial gene regulation and show how small regulatory RNAs can help control the expression of horizontally acquired virulence genes.

## Introduction

Horizontal gene transfer (HGT) plays a central role in bacterial evolution, enabling the acquisition of genes that confer new functions such as antibiotic resistance and virulence. However, incoming DNA can also disrupt cellular processes or impose metabolic costs and is therefore restricted by multiple layers of genome defence. The most frequent include restriction–modification systems, CRISPR-Cas, and cyclic oligonucleotide-based antiphage signalling systems (CBASS), among an expanding repertoire of defence systems [1].

Foreign DNA that evades initial restriction can be integrated into the genome but is frequently detrimental to host fitness [2]. In *Enterobacteriaceae*, foreign DNA is often more AT-rich than the host genome and is transcriptionally silenced by the nucleoid-associated protein H-NS, which binds to AT-rich regions and forms oligomeric complexes that repress transcription [3–8]. This process—termed xenogeneic silencing—enables bacteria to tolerate the presence of foreign genes without incurring their deleterious effects [2,9–11]. Deletion of *hns* is lethal in several pathogens [12–15] underscoring the role of H-NS in suppressing deleterious gene expression.

For horizontally-acquired genes to be retained under positive selection they must be expressed. Activation can occur through specific transcription factors that counteract H-NS-mediated silencing in response to environmental cues [15]. A broader anti-silencing strategy is used by bacteriophage T7 that encodes a protein (gene 5.5) that forms heterodimers with H-NS, reducing its DNA-binding activity and enabling phage transcription [16]. However, global interference with H-NS is likely to de-repress a broad range of largely detrimental genes and be costly to the host.

H-NS silencing is selectively enhanced at virulence loci by Hha, a small H-NS paralogue that lacks a DNA-binding domain but forms heterodimers with H-NS to increase its affinity for specific targets [17]. Hha appears to enhance H-NS activity on foreign genes, particularly at virulence-associated islands, without affecting the expression of H-NS-regulated genes in the core genome [17,18]. Structural and biochemical analysis of the Hha-H-NS heterodimer indicates that Hha coats the H-NS oligomerisation domain and presents a positive surface that increases DNA binding [18]. Modulation of Hha activity may therefore provide a mechanism to control xenogenic silencing without globally disrupting H-NS function.

Small regulatory RNAs (sRNAs) are central regulators of bacterial physiology, stress responses, virulence, and host–microbe interactions [19,20]. They are pervasive post-transcriptional regulators that reshape bacterial gene expression through sequence-specific interactions with target RNAs [21]. In *E. coli*, many sRNA interactions are facilitated by Hfq, and modulate translation, termination, and transcript stability enabling rapid adaptation to environmental signals. Recent work has also highlighted phage-encoded sRNAs as an important and emerging layer of phage–host regulation [22]. Even bacteriophage λ, one of the most intensively studied genetic systems, was recently shown to encode previously unrecognised Hfq-associated sRNAs that reprogram host physiology to promote phage propagation [23]. In pathogenic *E. coli* and *Vibrio cholerae*, prophage-encoded sRNAs modulate host gene expression and phage development [24,25], and prophage-encoded sRNA sponges reshape core bacterial sRNA regulatory networks [26]. Regulatory sRNAs are compact and portable, and appear to be well suited to mediating communication between phage and host cells to increase the fitness of one or both.

In Enterohaemorrhagic *Escherichia coli* (EHEC), horizontal gene transfer has introduced over 1.5 Mb of new genetic material, including 19 prophages that carry many key virulence factors [27,28]. We previously showed that these regions are rich in sRNAs, several of which contribute to virulence gene expression [24,26,29]. Here, we identify an sRNA, HnrS, encoded on prophages and present in up to nine copies in attaching and effacing *E. coli* strains that encode the locus of enterocyte effacement (LEE^+^). We show that HnrS directly represses *hha* translation, attenuating Hha-mediated silencing. HnrS modulates Hha-dependent phenotypes, including motility and type 3 secretion, and affects a subset of Hha-associated genes under T3SS-inducing conditions. These findings reveal an RNA-based counter-silencing mechanism encoded by prophages that relieves xenogenic repression in pathogenic *E. coli*.

## Results

### HnrS is a multicopy small RNA encoded on prophage

**Fig 1:**
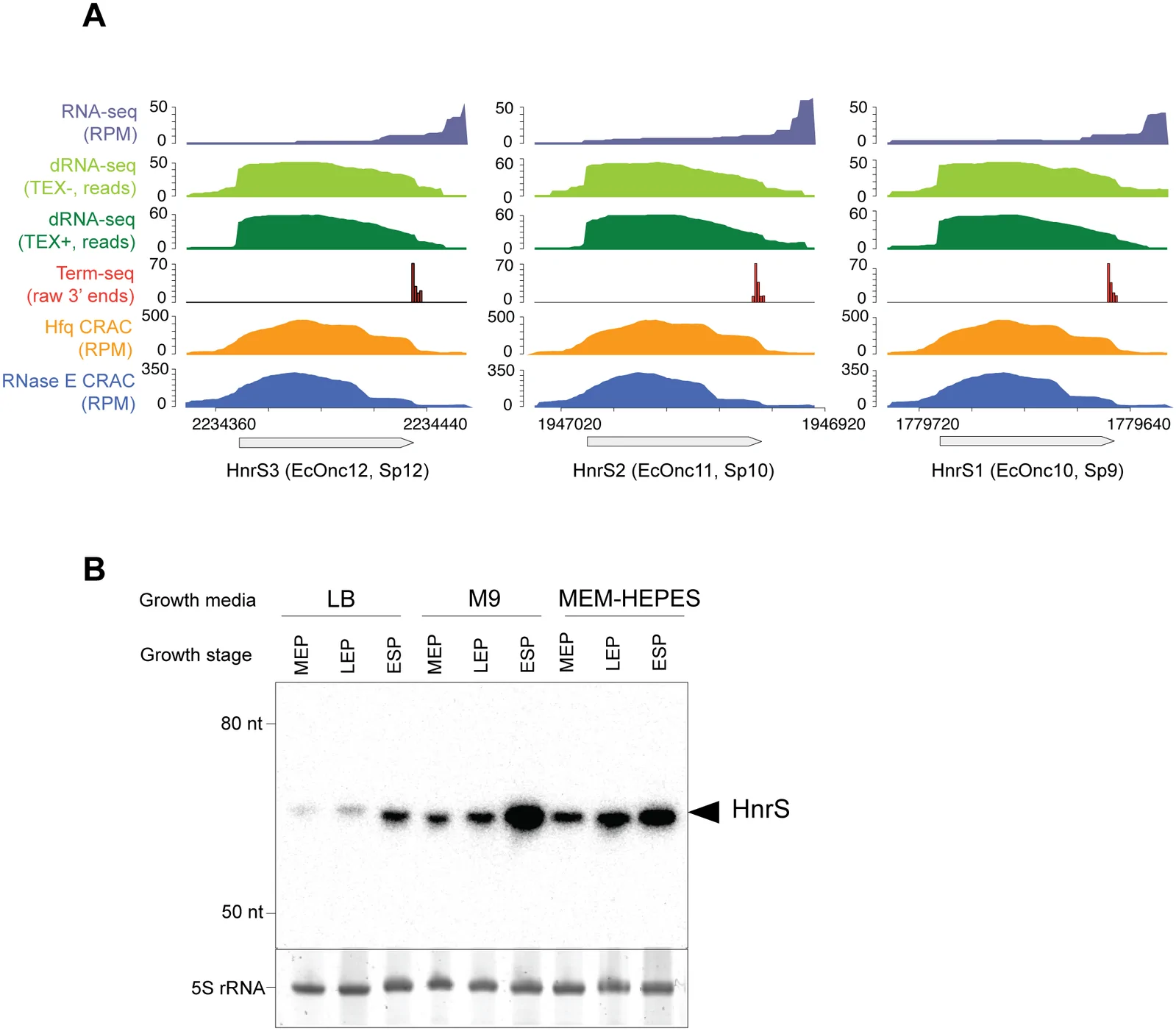
*E. coli* str. Sakai encodes three identical copies of sRNA HnrS on prophage. **A.** RNA end mapping, Hfq, and RNase E binding at HnrS. Total RNA-seq data from EHEC grown in MEM-HEPES (purple, [24]), dRNA-seq (light green, TEX-) [24], dRNA-seq (dark green, TEX+) [24], and Term-seq (red bar plot) [24], Hfq binding (orange, [21]), and RNase E binding (dark blue, [22]) for three genomic copies of HnrS in EHEC str. Sakai. Read abundance is indicated on the y-axis. Term-seq peaks correspond to transcription termination sites, while dRNA-seq reads highlight transcription start sites. Grey arrows indicate the position of HnrS with the systematic name and prophages (Sp9, Sp10, and Sp12) in brackets. **B.** Northern blot analysis of HnrS expression in LB, minimal M9, and MEM-HEPES media at mid-exponential (OD600 = 0.6), late exponential (OD600 = 1.0), and early stationary (OD600 = 1.8) phases.

In EHEC strains horizontally-acquired pathogenicity islands encode the majority of genes required for virulence. In our previous analysis of the sRNA interactome in EHEC str. Sakai we identified several sRNAs within the pathogenicity islands (S-loops) that had abundant interactions with mRNAs [29], suggesting that they may contribute to virulence. Among the most abundant were three copies of a sRNA with 100% sequence identity that were designated EcOnc10, EcOnc11, and EcOnc12 and encoded within the prophages Sp9, Sp10, and Sp12 respectively. We have renamed this sRNA HnrS for Hha-negative regulatory small RNA (described below). We had previously identified these sRNAs through their Hfq-binding [26] indicating that they are Hfq-associated sRNA (Fig 1A). To precisely map the 5’ and 3’ ends of the sRNAs we analysed dRNA-seq and Term-seq data for EHEC str. Sakai [24]. Each copy had the same 5’ and 3’ end and produced a 66-nt sRNA that is terminated by an intrinsic terminator (Fig 1A). Northern blot analysis supported transcription of a 66-nt sRNA and indicated that it accumulates during early stationary phase in both T3SS-inducing media (supplemented MEM-HEPES) and minimal media (M9) (Fig 1B), similar to the Stx phage sRNA, StxS [23].

**Fig 2:**
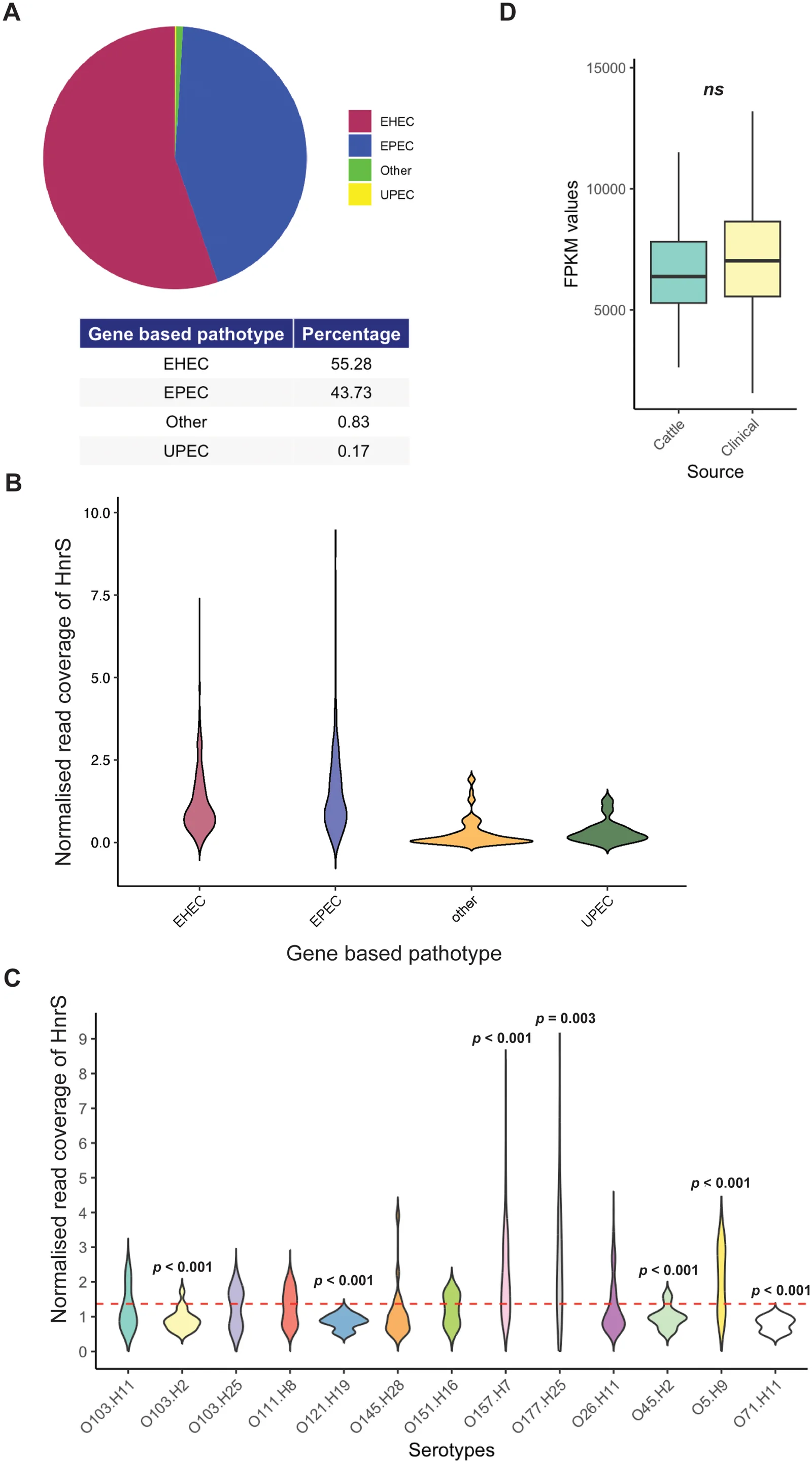
Distribution and coverage of *hnrS* across *E. coli* pathotypes and serotypes. **A.** Coverage of *hnrS* across different *E. coli* pathotypes, including EHEC, EPEC, UPEC, and others where values were normalised to six conserved housekeeping genes (*arpA*, *trpA*, *uidA*, *rrl*, *tspE4*, and *fyuA*) as a reference. **B.** Violin plot representing maximum *hnrS* coverage across EHEC, EPEC and UPEC strains. **C.** Distribution of *hnrS* read coverage across STEC serotypes. Asterisks above the violins indicate statistically significant deviations from the overall median, as determined by one-sample t-tests. **D.** Box plot of *hnrS* coverage (FPKM) in O157:H7 isolates from human and cattle sources.

To understand whether HnrS is found in other *E. coli* pathotypes, we searched for copies within 3,230 *E. coli* genome sequences available on NCBI that were pathotyped by looking for characteristic virulence genes (Methods & Supplementary Information) [30,31]. The majority of strains that carried a copy of *hnrS* were enterohaemorrhagic *E. coli* (EHEC) or enteropathogenic *E. coli* (EPEC) pathotypes (99.01%), with a small fraction of uropathogenic (0.82%) and unclassified (1.17%) *E. coli* strains (Fig 2A). Shiga toxin genes (*stx1* and *stx2* that partly characterise EHEC) were enriched in the *hnrS*-encoding strains (*p* < 0.0001), as was the LEE that facilitates attaching and effacing lesion formation in EHEC and EPEC (*p* < 0.0001, Fishers exact test).

Three copies of HnrS are encoded on prophages in EHEC str. Sakai. To assess variation in gene dosage across strains, the relative copy number of *hnrS* was estimated based on normalised sequencing read depth (Fig 2A-2B). Specifically, the coverage of HnrS was normalised against the mean coverage of six conserved housekeeping genes (*arpA*, *trpA*, *uidA*, *rrl*, *tspE4*, and *fyuA*). The resulting values represent relative read coverage that approximates copy number within the genome. The median relative coverage for *hnrS* among O157:H7 strains was approximately 2-fold, with the highest exceeding 7-fold in one isolate (Fig 2C). Among EHEC serotypes, O177:H25 and O157:H7 exhibited a significantly higher *hnrS* coverage, with an O177:H25 isolate encoding 9-fold more *hnrS* than the housekeeping gene controls. Serotype O157:H7 is the most common EHEC serotype associated with human infection. To determine whether a higher *hnrS* copy number is associated with human infection, we compared the read depth (FPKM [Fragments per kilobase per million mapped reads]) within a collection of EHEC genomes from human infections and the natural reservoir host, cattle, [32]. Although the difference in *hnrS* coverage between human and bovine isolates did not reach statistical significance, there is a trend toward higher copy number in human isolates. (Fig 2D).

Recent work has identified bacteriophage sRNAs that promote phage survival [23,25]. To understand if *hnrS* is found in prophage in other EHEC and EPEC strains, we used PHASTER to identify prophage regions in a subset of strains. In each EPEC and EHEC strain, a single copy of *hnrS* was encoded within the late genes of an independent prophage element (S1 Fig). These results indicate that *hnrS* is acquired by insertion of prophages into the genome, and multiple copies arise from the acquisition of multiple prophages.

### HnrS represses the xenogeneic DNA silencer, Hha

The acquisition of multiple copies of *hnrS* on phage suggests that the sRNA contributes to phage or host fitness. Using our previously published sRNA interactome data (RNase E-CLASH) [29], we identified target mRNAs for HnrS. The most abundant interaction for HnrS was with the toxin-antitoxin (TA) system, *mazEF* (129-hybrids, FDR = 0). The *mazEFG* operon encodes the TA system followed by *mazG*, which encodes a nucleoside triphosphate pyrophosphohydrolase [33]. HnrS has perfect complementary with the last 15-nt of the *mazF* coding sequence (S2A Fig) and we confirmed that HnrS interacts with the *mazF* coding sequence *in vitro* using EMSA (S2B-S2C Fig).

Despite this interaction, we were unable to detect functional consequences of HnrS on *mazEFG*. Overexpression of *mazEFG* was toxic to *E. coli* str. MG1655, but co-expression of HnrS had no appreciable effect on MazEFG toxicity (S3A Fig). MazEF has been suggested to confer phage resistance [33–35]. Deletion of *mazEFG* in *E. coli* K12 str. MG1655 did not confer sensitivity to P1, T4, λ, or a V5-like phage, indicating that HnrS regulation of *mazEF* would not alter sensitivity to these phages through *mazEFG* (S2D Fig). The HnrS interaction has 15-nt of complementary with the last 5 codons of *mazF* suggesting the interaction would not function to block translation initiation. We hypothesised that it might recruit RNase E to promote *mazEFG* degradation. To understand if HnrS might direct processing of the *mazFG* intergenic region or regulate downstream *mazG*, we constructed a *mazFG* GFP translational fusion and monitored fluorescence in the presence or absence of HnrS. MazG’-GFP was only modestly reduced (S3B Fig). These data indicate that although HnrS has abundant interactions with *mazF* in our RNase E-CLASH data, we found no evidence that it significantly affects MazEFG function under the conditions tested.

**Fig 3:**
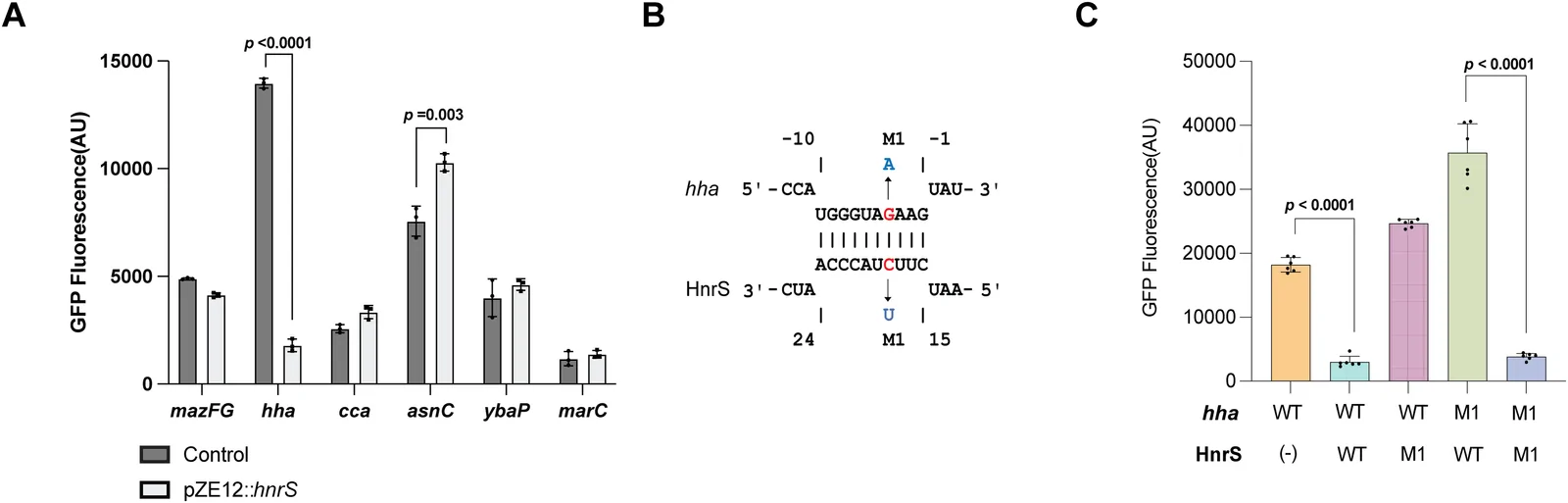
*In silico* and experimental validation of HnrS–mRNA interactions. **A.** Target mRNA–sfGFP fluorescence in the presence of HnrS or scrambled control plasmid (pJV300) for the first six genes in the RNase E-CLASH sRNA interactome. **B.** Compensatory point mutations (blue) were introduced in *hha* or HnrS at the M1 seed region to disrupt predicted base-pairing. The sequence AGAAG upstream of the *hha* coding region corresponds to the ribosome binding site (RBS). Numbers above *hha* indicate distance from the start codon and numbers below HnrS indicate distance from the 5’ end. **C.** Fluorescence of *hha*–sfGFP was measured in the presence of HnrS wild type or mutant variants, with restoration of interaction by complementary mutations confirming direct pairing. Data represents mean GFP intensity from three biological replicates with two technical replicates each.

To identify functional targets of HnrS we constructed GFP translational fusions to the remaining five HnrS-mRNA interactions identified by RNase E-CLASH (FDR < 0.2 or hybrid count>2). Two mRNAs were significantly regulated by HnrS, the transcription factor AsnC was upregulated (1.32-fold, *p* = 0.03) and the xenogeneic silencer Hha was repressed 10-fold (*p* < 0.0001) (Fig 3A-3B). Given the strong repression of *hha*, and coordinate regulation in our RNA-seq data (described below), we focussed on HnrS regulation of *hha*. To confirm that HnrS is a direct repressor of Hha we introduced compensatory point mutations into the HnrS and *hha* seed sequence. Single point mutations in the sRNA or mRNA seed disrupted repression and introduction of compensatory mutations into both sRNA and mRNA restored repression indicating that regulation was dependant on direct base-pairing of HnrS and *hha* RNAs (Fig 3C).

Hha forms a complex with H-NS and promotes silencing of predominately horizontally-acquired genes and virulence genes in *E. coli* and *Salmonella* [17,18,36]. Our data indicate that the prophage-encoded sRNA HnrS represses Hha, suggesting that it acts to counter xenogeneic silencing.

### HnrS represses motility in commensal and enterohaemorrhagic *E. coli*

**Fig 4:**
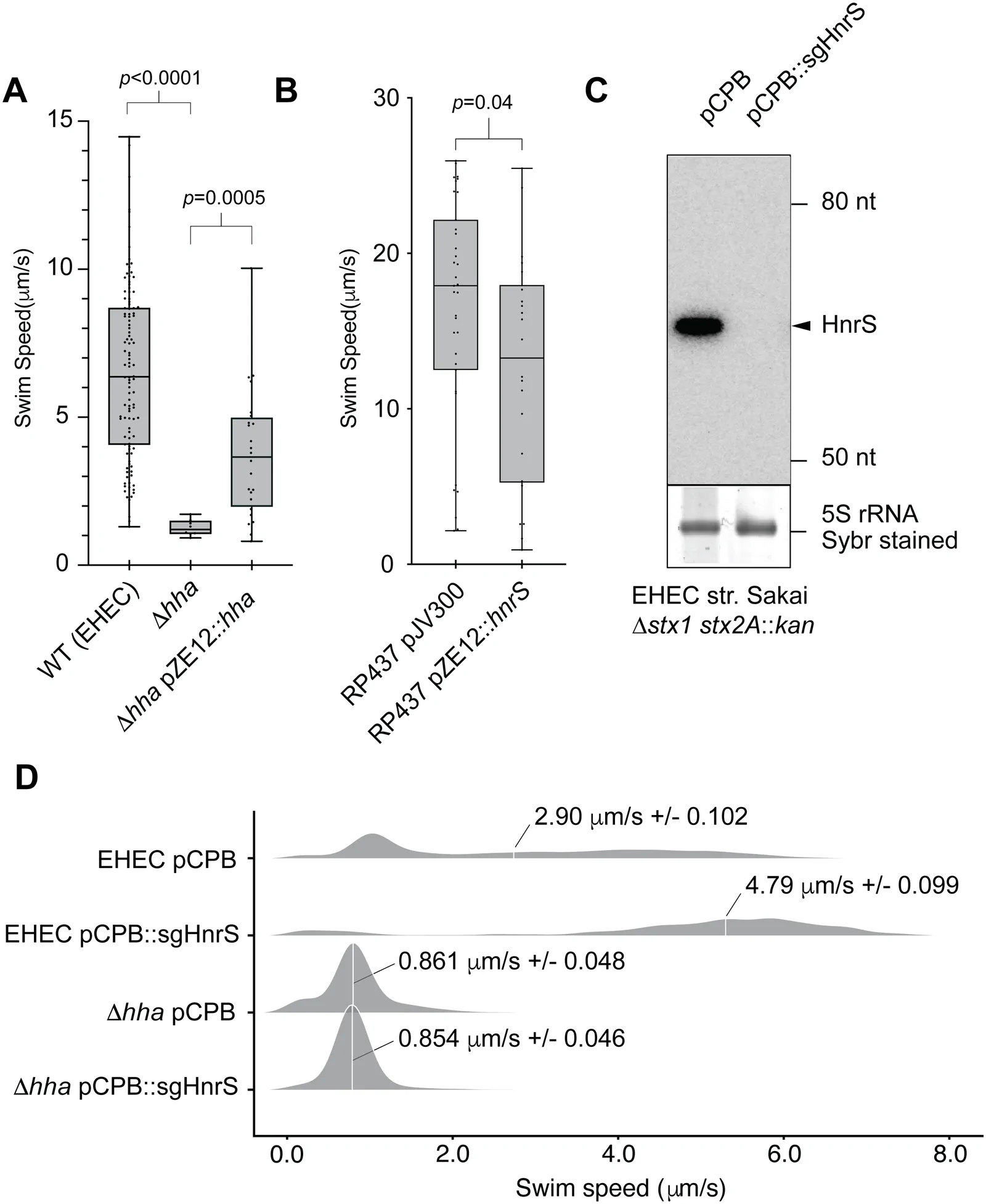
HnrS knockdown and ∆*hha* exhibit altered motility. **A.** Single-cell swimming speed of EHEC str. Sakai wild type, ∆*hha* with empty vector (pBR322), and ∆*hha* complemented with pBR322::*hha*. Cells were imaged in tunnel slides under phase-contrast microscopy (WT n = 118, ∆hha n = 11, complemented n = 30). **B.** Single-cell speed assay for hypermotile commensal *E. coli* RP437 wild type (does not encode *hnrS*), RP437 with empty vector (pJV300), and RP437 carrying pZE12::*hnrS*. **C.** Northern blot detecting HnrS expression in wild type and HnrS CRISPRi knockdown strains (pCPB vector), with RNA harvested at early stationary phase (OD_600_ = 1.8). **D.** Swim speed measurements for EHEC str. Sakai stx(-) strains with vector control (pCPB) and HnrS knockdown (pCPB::sgHnrS), and the isogenic ∆*hha* strains. Mean swim speeds are indicated +/- standard error. Each condition represents data from 4 biological replicates (EHEC pCPB total cells *n* = 3183, pCPB::sgHnrS *n* = 983, ∆*hha* pCPB n = 630, ∆*hha* pCPB::HnrS *n* = 1153). Statistical significance was calculated using a Mann-Whitney U test.

Hha positively regulates motility in *E. coli* and *Salmonella* through activation of the master flagellar regulator FlhDC [37,38]. We initially looked to quantify Hha regulation of motility in EHEC str. Sakai. Swim speed was measured for the wild type, ∆*hha*, and the complemented mutant (∆*hha* pBR322::*hha*) using phase contrast microscopy to track motility (Fig 4A). Consistent with earlier reports in *E. coli* and *Salmonella*, deletion of *hha* dramatically reduced swim speed in EHEC str. Sakai (4.7-fold, *p* < 0.0001) and was restored in the complemented background.

To assess whether HnrS is able to repress motility, a HnrS expression construct (pZE12::*hnrS*) was moved into the hypermotile commensal *E. coli* str. RP437 (that lacks the *hnrS-*encoding prophages). Expression of HnrS in the commensal background significantly reduced motility compared to a scrambled sRNA control (Fig 4B), consistent with HnrS repression of *hha*.

To simultaneously knockdown expression of all three copies of HnrS in EHEC we used CRISPR interference (CRISPRi; catalytically inactive dCas9, expressed from a plasmid construct). HnrS was not detectable by Northern blot at early stationary phase indicating efficient knockdown in the CRISPRi strains (Fig 4C). Knockdown of HnrS in EHEC significantly increased motility (1.64-fold, *p* = 0.03), consistent with our overexpression results in commensal *E. coli* str. RP437 (Fig 4D).

To confirm that HnrS repression of motility acted through repression of *hha,* we performed an epistasis experiment where HnrS was knocked down in the EHEC ∆*hha* background and swim speeds were measured (Fig 4D). As earlier, the EHEC ∆*hha* strain was non-motile and knockdown of HnrS did not significantly increase the swim speed (*p* = 0.89), consistent with HnrS repressing motility through *hha*.

### HnrS controls T3SS in EHEC

**Fig 5:**
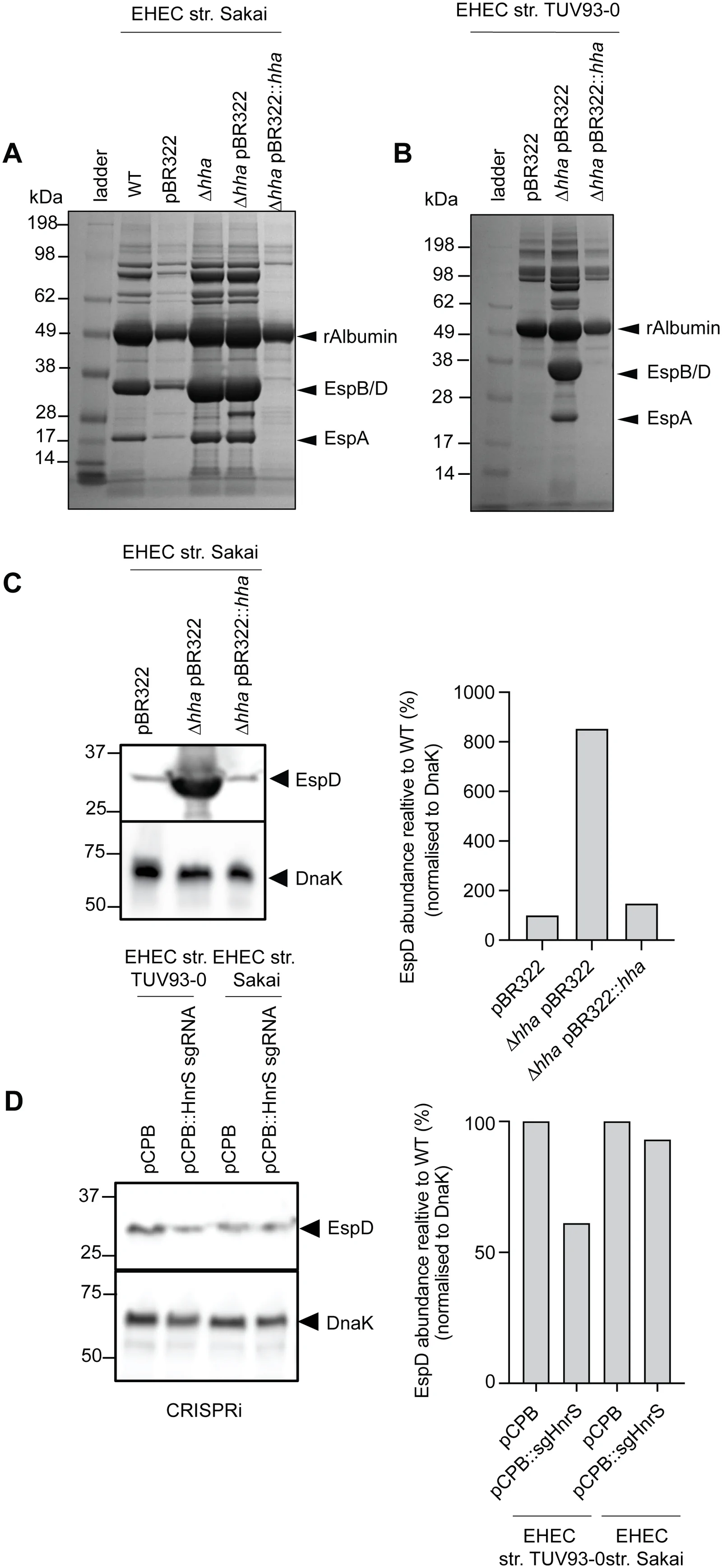
T3SS secretion profiles of *hha* and HnrS mutants in EHEC Sakai and TUV93-0 strains. **A.** Coomassie-stained SDS-PAGE gels showing secreted protein profiles. **Left panel:** EHEC Sakai stx(-) wild type, wild type with pBR322, ∆*hha*, ∆*hha* with pBR322, and ∆*hha* complemented with pBR322::*hha*. **B.** EHEC TUV 93-0 stx(-) wild type, ∆*hha*, and ∆*hha* complemented with pBR322::*hha.* **C.** Western blot analysis of EspD in secreted fractions and DnaK in whole-cell samples. (left) EHEC str Sakai wild type with pBR322 (pBR322), ∆*hha* with pBR322, and ∆*hha* complemented with pBR322::*hha*. (right) Densitometry analysis of the EspD bands from these blots is shown alongside each Western blot. **D.** Western blot analysis of EspD and DnaK after HnrS knockdown in EHEC. (left) Strain backgrounds are indicated above. Empty vector pCPB (pCPB), TUV 93-0 with pCPB::sgHnrS (lane 2), Sakai with pCPB (lane 3), and Sakai with pCPB::sgHnrS (lane 4). Statistical significance was assessed by unpaired *t*-test.

Expression of the T3SS is essential for EHEC colonisation of both ruminants and humans, and is silenced by Hha-H-NS [39–42]. Given that 99.01% of genomes that encode *hnrS* are also LEE + , we speculated that a major selective pressure for maintaining multiple copies of HnrS may be de-repression of the LEE to promote colonisation. We first tested the effect of deleting *hha*, reasoning that this would define the expected consequence of relieving Hha-dependent silencing in our strain backgrounds. We used both EHEC str. Sakai, which has relatively low basal type 3 secretion, and the high-secretion strain TUV93–0 to increase the dynamic range for detecting changes in T3SS output. Deletion of *hha* in both strain backgrounds resulted in strong de-repression of T3SS that was restored to wild type levels in the complemented strain (Fig 5A).

To determine whether Hha acts at the level of LEE promoter activity, we next measured GFP transcriptional fusions to the LEE1 (*ler*), LEE5 (*tir*), and LEE4 (*sepL*) promoters in the Δ*hha* background. These experiments were designed to test the Hha-dependent arm of the HnrS pathway, rather than direct regulation of LEE promoters by HnrS (S4 Fig). Consistent with earlier work [43], deletion of *hha* de-repressed the LEE1 and LEE5 promoters and was restored to wild type in the *hha* complementation strain. The LEE4 promoter was not de-repressed in the *hha* mutant consistent with post-transcriptional repression of *sepL* translation [44].

Having established that loss of Hha de-represses LEE promoter activity and T3SS output, we next asked whether depletion of HnrS has the reciprocal effect predicted by the model. Because the three *hnrS* copies are identical, we used CRISPRi to simultaneously knock down all copies in Sakai and TUV93–0. Total secreted protein levels were reduced in strains carrying the dCas9 plasmid, including vector controls, so we quantified the secreted T3SS translocon protein EspD by immunoblotting. EspD was de-repressed (8.53-fold) in the EHEC str. Sakai ∆*hha* background and restored to wild type in the complemented strain (Fig 5C). HnrS knockdown significantly reduced EspD abundance in both TUV93–0 (1.7-fold) and Sakai (1.1-fold), although the effect was stronger in the high-secretion TUV93–0 background (Fig 5D).

Together, these data support a model in which HnrS promotes T3SS expression by repressing *hha*: removal of Hha de-represses LEE promoter activity and T3SS secretion, whereas depletion of HnrS reduces EspD secretion, consistent with increased Hha-dependent silencing.

### HnrS increases pedestal formation on epithelial cells

**Fig 6:**
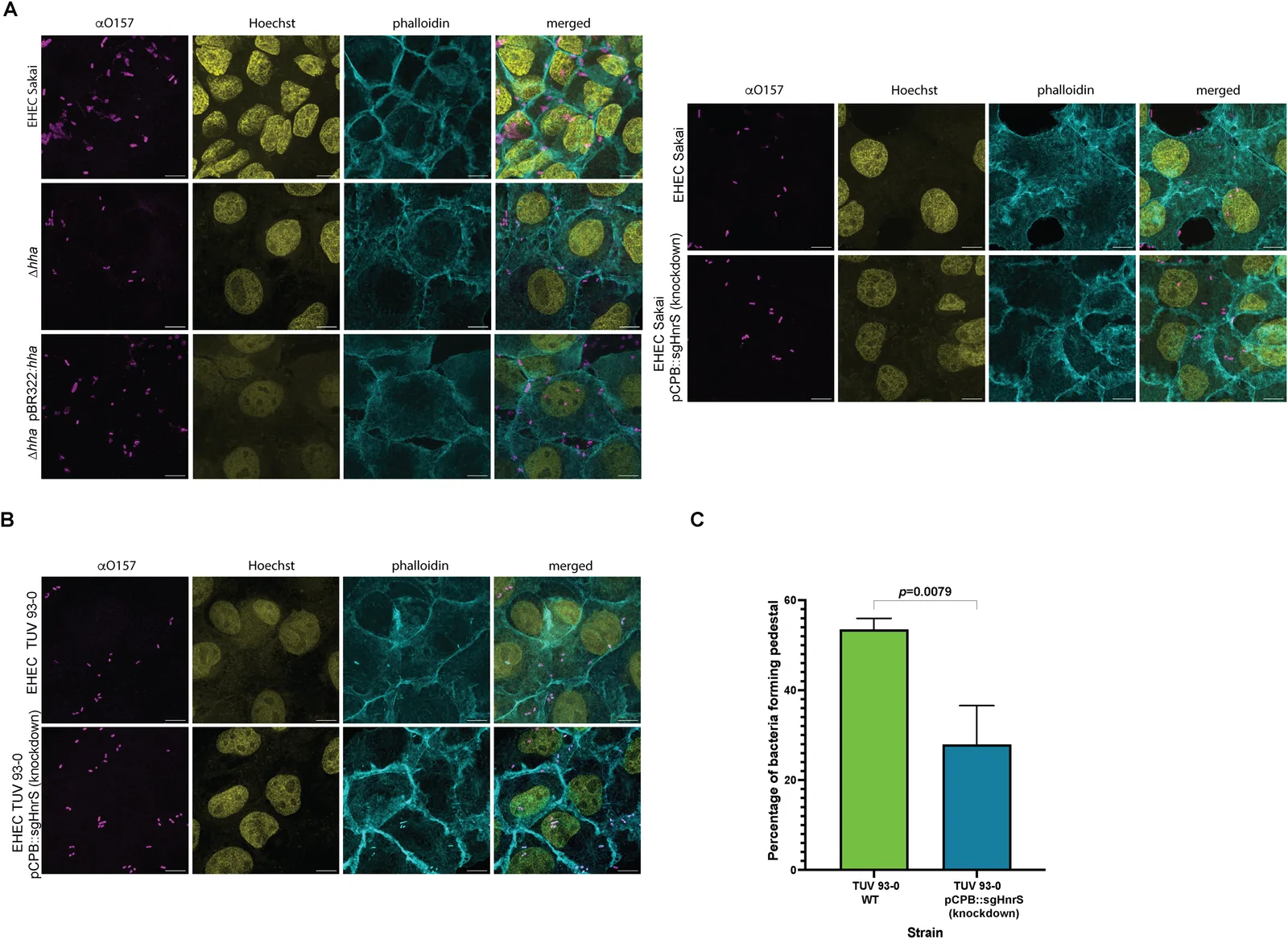
Immunofluorescence analysis of EBL cells infected with EHEC ∆*hha* and HnrS mutants. **A. Left panel:** EBLs were infected with EHEC Sakai strains carrying pBR322 (wild type), ∆*hha* with pBR322, and the complemented ∆*hha* strain with pBR322::*hha* (MOI 100). Bacteria including flagella (magenta) were visualized using anti-O157 antibodies with Alexa Fluor 568 secondary, nuclei (yellow) were stained with Hoechst 33342, and actin filaments (cyan) were visualized with Phalloidin-FITC. Merged images show co-localization of all stains. Wild type shows intact flagella, ∆*hha* lacks flagella, and the complemented strain restores flagella expression. **Right panel:** EBLs were infected with EHEC Sakai carrying either pCPB (empty vector) or pCPB::sgHnrS (HnrS knockdown) at MOI 100. **B.** EBLs were infected with EHEC TUV 93-0 carrying either pCPB (empty vector) or pCPB:: sgHnrS (HnrS knockdown) at MOI 100. C. The percentage of bacteria inducing actin polymerisation at adhesion sites was measured from fluorescence images.

Our results suggest that HnrS expression promotes colonisation through decreased motility and increased T3S (attachment). To assess the impact of HnrS and *hha* on host cell adhesion, we incubated the *hha* strains and *hnrS* knockdowns with embryonic bovine lung cells (EBLs), a bovine epithelial cell line that has been used extensively to quantify attachment and actin pedestal formation of EHEC [45–48]. Despite de-repression of the T3SS that is required for attachment and colonisation, we did not observe a statistically significant difference in attachment with either the *∆hha* or *hnrS* knockdown strains in the low T3S secretion strain Sakai backgrounds (Fig 6A). We next assessed adhesion of the *hnrS* knockdown in the high secretion strain, TUV93–0. Similar to strain Sakai, there was no statistically significant difference in EHEC adhesion to EBL cells (Fig 6B), but the *hnrS* knockdown had significantly less actin-rich pedestal formation – a key step in T3S-dependant intimate attachment (Fig 6C). Quantification of actin-rich pedestals indicated a 1.91-fold (*p* = 0.0079) reduction in pedestal formation at 5 hours post-infection. These data suggest that HnrS promotes earlier pedestal formation in EHEC.

### HnrS influences expression of prophage-encoded virulence genes and T3SS regulators

**Fig 7:**
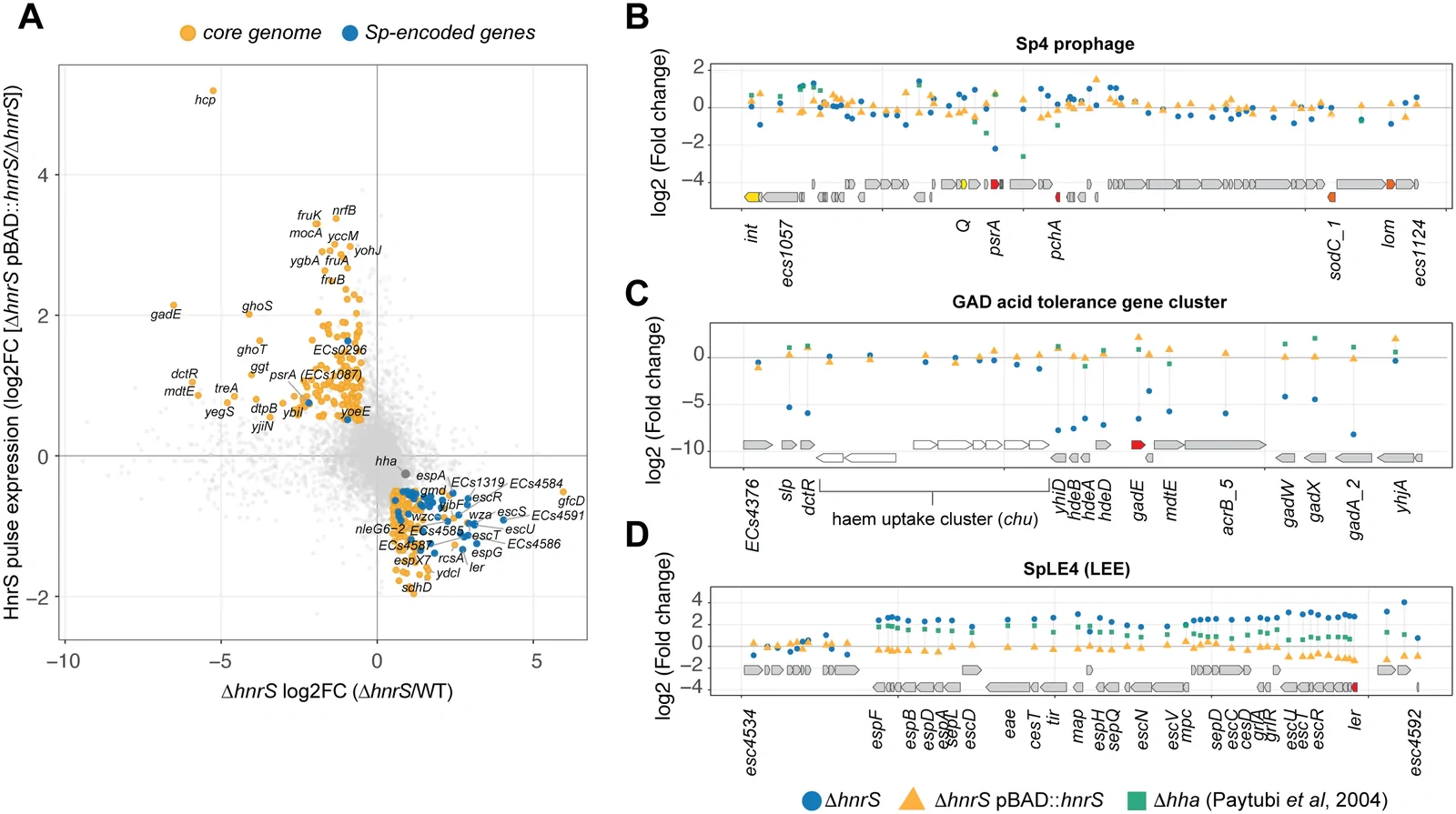
HnrS regulates virulence genes within horizontally-acquired prophages. **A.** Differential gene expression in EHEC str. Sakai ∆*hnrS* and after HnrS pulse expression (∆*hnrS* pBAD::*hnrS*). Genes that have reciprocal regulation in the ∆*hnrS* strain and after HnrS pulse expression (reciprocal |log2FC| > 0.5 and FDR<=0.05 in both conditions) are coloured (blue or yellow). Genes with reciprocal regulation that are encoded within horizontally-acquired prophage regions (Sp-loops) are indicated in blue, and all other genes are colured yellow. **B-D**. Differential gene expression with the Sp4 prophage **(B)**, glutamate-dependant acid tolerance (GAD) gene cluster **(C)**, and the Locus of Enterocyte Effacement (LEE, SpLE4) (**D**). Relative gene expression values are shown for ∆*hnrS*/WT (∆*hnrS*, blue circle), ∆*hnrS* pBAD::*hnrS* (∆*hnrS* pBAD::*hnrS*/∆*hnrS*, yellow triangles), and ∆*hha* (∆*hha*/WT, green squares) [38]. Grey arrows indicate the organisation of genes within the region and select genes are labelled below. Select transcriptional regulators of the LEE (red), phage regulators (yellow), and virulence genes (orange) are indicated.

To define the regulatory scope of HnrS, we generated a clean chromosomal deletion of all three *hnrS* loci (*ΔhnrS1–3*; hereafter Δ*hnrS*) in the EHEC str. Sakai background using CRISPR–Cas9 genome editing. Earlier phenotypic analyses were performed in the isogenic Sakai ∆*stx1 stx2A*::*kan* derivative. For transcriptomic profiling and complementation, we used the Shiga toxigenic parental Sakai strain to allow controlled plasmid-based expression of HnrS. Deletion of all three copies was confirmed by Northern blot (Fig 7A).

Wild-type, Δ*hnrS*, and complemented strains were grown under T3SS-inducing conditions (MEM-HEPES). To enrich for primary regulatory effects, HnrS was pulse-expressed for 15 minutes prior to RNA harvest in the complemented strain. Differential expression analysis identified 108 genes significantly up- or down-regulated in the Δ*hnrS* strain relative to wild type (log2 fold change >2, adjusted p < 0.05; Fig 7A). Transcript levels for *hha* were elevated in the Δ*hnrS* strain (log2FC[∆*hnrS*/WT]=0.940, FDR = 2.0x10^9^) and partially complemented after pulse expression of HnrS but did not reach statistical significance (log2FC[complement/∆*hnrS*]=-0.268, FDR = 0.165)(S5 Fig), consistent with direct repression of *hha* by HnrS.

We next examined horizontally-acquired prophages (termed Sp-loops in EHEC strain Sakai) to assess HnrS regulation. Many specific regions of the Sp-loops were differentially regulated and this partly correlated with genes that had previously been found to be regulated in an EHEC *hha* deletion strain (S6 and S7 Figs) [42]. Genes within the Mu-like prophage (Sp18) were strongly repressed in the ∆*hnrS* strain but were not complemented by HnrS pulse expression. Examination of read counts within this region suggests that the prophage has excised during construction of the ∆*hnrS* strain (S8 Fig).

We found that the LEE (SpLE4) is upregulated in the ∆*hnrS* deletion and partly repressed by pulse-expression of HnrS (Fig 7D), in contrast to our results demonstrating repression of EspD after HnrS knockdown. This result prompted us examine the transcriptome for additional LEE regulators that were differentially expressed. Among the prophage-encoded regions, several genes were down-regulated in the ∆*hnrS* background and had reciprocal regulation after HnrS pulse expression (Figs 7A and S6 and S7). These included the Ogr/Delta-like P4 phage activator *ecs0296* (Sp2) and the prophage-encoded T3 secretion regulator *psrA* (*ecs1087*, Sp4) that were repressed in the ∆*hnrS* strain and upregulated by HnrS pulse expression (Figs 7B and S6 and S7). PsrA activates the T3SS repressor *gadE* after attachment and pedestal formation [45], and *gadE* was also strongly repressed in the ∆*hnrS* strain and upregulated after HnrS pulse expression (Fig 7C), consistent with activation of the PsrA/GadE regulatory cascade that represses the LEE. While *psrA* and *gadE* are strongly upregulated after pulse expression, we were not able to identify complementarity between HnrS and the *psrA* mRNA that would indicate stabilisation by HnrS (e.g.,: 5’ end protection), suggesting that the regulation is indirect.

The data suggest that HnrS controls an incoherent feed-forward loop that positively regulates the LEE through repression of *hha*, and negatively regulates the LEE through activation of PsrA/GadE.

### HnrS influences expression of nitrate and nitric oxide metabolism genes

To assess whether specific functional categories were overrepresented among differentially expressed genes, we performed gene ontology (GO) enrichment analysis. This analysis identified nitrogen metabolism–associated processes as significantly enriched in wild type relative to Δ*hnrS* (S9 Fig). Guided by this enrichment signal, we examined the underlying loci and observed coordinated changes in several operons involved in anaerobic nitrate and nitrite respiration, including *nirBD*, *nrfABCD*, and components of the *nap* and *nar* systems (S10A Fig). Expression of *hcp*, encoding a nitric oxide reductase, was reduced in Δ*hnrS* and the most strongly increased gene following HnrS pulse expression (Fig 7A).

Translational GFP reporter assays did not detect direct regulation of these genes by HnrS (S10B Fig), indicating that the observed transcriptional changes are likely indirect. Comparison with previously published Δ*hha* transcriptomic data [42] revealed partial overlap between several HnrS-dependent nitrate/nitrite metabolism genes and the Hha regulon (S11 Fig). These findings support a model in which HnrS influences expression of a defined subset of metabolism-associated genes primarily through repression of *hha*.

## Discussion

Xenogenic silencing mediated by H-NS enables bacteria to tolerate horizontally acquired DNA while limiting inappropriate gene expression. In enteric pathogens, this repression is enhanced at virulence loci by the H-NS paralogue Hha, which strengthens silencing of horizontally acquired regions [17,18,49]. Here, we identify a prophage-encoded small RNA, HnrS, that directly represses *hha* translation through base-pairing at its ribosome binding site. By reducing Hha levels, HnrS attenuates Hha-dependent silencing and modulates expression of virulence-associated loci.

Our genetic and molecular data identify *hha* as a direct and functionally relevant target of HnrS. Compensatory mutagenesis confirms base-pairing at the *hha* 5′ UTR, and altering HnrS levels modulates Hha-dependent phenotypes, including motility and type III secretion. These findings support a model in which HnrS functions as an RNA-based counter-silencer that selectively modulates Hha.

Counter-silencing strategies are common among horizontally acquired virulence loci, which frequently encode transcription factors such as Ler or VirB that locally relieve H-NS repression [15,50]. Bacteriophages have also evolved strategies to overcome xenogeneic silencing [51], including T7 gp5.5, which disrupts higher-order H-NS oligomerisation [16,52], T4 Arn, which acts as a DNA mimic to interfere with H-NS DNA binding [53], T4 MotB, which alters H-NS-dependent gene regulation through nucleoid remodelling [54,55], and phage LUZ24 gp4, which antagonises the H-NS-family silencer MvaT in *Pseudomonas* [56]. These examples act through proteins that directly target H-NS-family proteins or remodel their interactions with DNA. In contrast, HnrS provides an RNA-based counter-silencing strategy: rather than directly inhibiting H-NS, HnrS represses translation of the H-NS paralogue Hha, which selectively enhances H-NS silencing at virulence-associated loci. To our knowledge, HnrS therefore represents the first phage-encoded sRNA shown to antagonise xenogeneic silencing.

This distinction between H-NS inhibition and Hha repression may be particularly relevant for prophages. Many characterised phage anti-H-NS systems are encoded by lytic phages, where transient disruption of host silencing during infection may be tolerated or advantageous [51]. Prophages, however, must persist within the bacterial chromosome over many generations, and broad disruption of H-NS would likely impose fitness costs on the lysogen. Targeting Hha rather than H-NS may therefore provide a more selective strategy, allowing HnrS to attenuate Hha-enhanced silencing at horizontally acquired virulence loci while preserving much of the core H-NS regulatory network [17,18]. Selection for *hnrS* may directly benefit the prophage, but may also improve fitness of the lysogen through tuned expression of horizontally acquired virulence loci, including LEE-associated genes, non-LEE effectors, and prophage-encoded regulators. In this model, HnrS acts as a compact, multicopy regulatory module that enables prophages to selectively tune Hha-dependent xenogeneic silencing in the lysogen.

**Fig 8:**
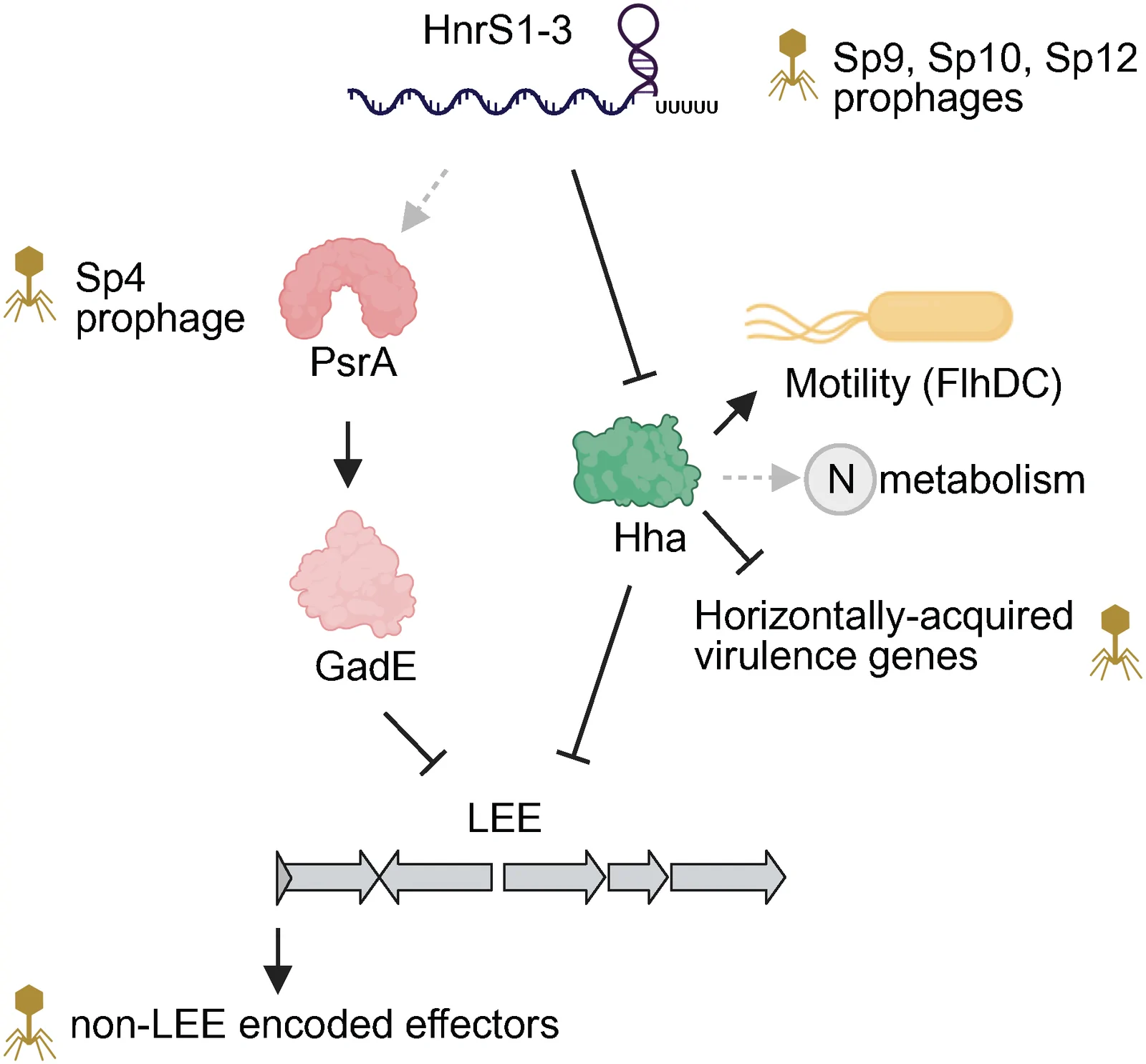
Model of HnrS regulation in EHEC. Solid black lines indicate experimentally verified regulatory interactions, and grey dashed lines indicate differential expression in the ∆*hnrS* and complemented strain alone (potentially indirect regulation). Arrows indicate positive regulation and bar-headed lines indicate repression [69].

Transcriptomic profiling of a Δ*hnrS* mutant revealed differential expression of genes under T3SS-inducing conditions. As expected, *hha* transcript levels increased in the absence of HnrS and were reduced upon pulsed HnrS expression, consistent with direct repression. However, HnrS regulation of the T3SS appears to be more complex than a simple linear HnrS–Hha–LEE pathway. Deletion of *hha* strongly de-repressed LEE promoter activity and EspD secretion, confirming that Hha represses the T3SS in our strain backgrounds. In contrast, HnrS depletion produced a more modest reduction in EspD secretion. This difference is consistent with the transcriptomic data, which suggest that HnrS participates in an incoherent feed-forward loop: HnrS promotes LEE expression through repression of *hha*, but also activates the PsrA/GadE pathway, which can negatively regulate the LEE (Fig 8). HnrS may have opposing effects on LEE transcription depending on the regulatory state of the cell, while still promoting T3SS under the secretion-inducing conditions tested here.

One possibility is that these regulatory arms operate at different stages of host interaction. PsrA/GadE are activated after EHEC attachment to EBL cells or red blood cells [45,57], suggesting that HnrS-dependent repression of *hha* and activation of PsrA/GadE may be temporally separated during colonisation. Under our *in vitro* T3SS-inducing conditions, these pathways may be active at the same time, dampening the magnitude of the HnrS knockdown phenotype relative to the Δ*hha* mutant.

Within the core genome, differentially expressed genes included loci involved in anaerobic nitrate and nitrite respiration and nitric oxide detoxification. These genes were not directly regulated by HnrS in translational reporter assays and partially overlapped with previously defined Hha-regulated loci, supporting an indirect mechanism mediated through Hha repression. In EHEC, nitrates trigger maturation of the T3SS by acting as a checkpoint between the basal body and needle filament assembly [58] providing a layer of coordination between nitrate availability and T3SS. Nitrates and nitrites also serve as an important terminal electron acceptors for anaerobic respiration during gastrointestinal colonisation [58,59] and hint at a role for HnrS in promoting expression of genes required for gastrointestinal colonisation.

HnrS is encoded on prophages and occurs in multiple copies in many LEE-positive *E. coli* strains. Repeated acquisition of *hnrS* through independent prophage insertions likely increases the dosage-dependent modulation of Hha levels. The strong enrichment of *hnrS* among attaching and effacing pathotypes suggests that de-repression of Hha-mediated silencing may provide a selective advantage to the host in LEE+ strains. Through de-repression of horizontally-acquired virulence loci (including non-LEE encoded effectors and transcriptional regulators within prophage, S6 and S7 Figs), HnrS may also provide a selective advantage for maintenance of the prophage.

Although we recovered a strong interaction between HnrS and *mazF* in our RNase E-CLASH data and were able to demonstrate an RNA–RNA interaction *in vitro*, we did not detect a measurable functional for this interaction under the conditions tested. It remains possible that this interaction contributes to phage or host fitness in contexts not captured here. However, repression of *hha* represents the primary detectable regulatory activity of HnrS under the conditions examined.

Our data demonstrate that HnrS is a prophage-encoded small RNA that directly represses the xenogenic silencer Hha, thereby modulating expression of specific virulence-associated loci. These findings reveal an RNA-based mechanism by which prophage modulate host silencing systems, providing a strategy to counter xenogenic silencing.

## Methods

### Bacterial strains and growth conditions

Bacterial strains, primers, plasmids and probes used for this study are listed in S1 Table. *E. coli* was routinely cultured at either 30°C or 37°C in LB broth, minimal M9 broth, MEM-HEPES (Sigma M7278) supplemented with 0.1% glucose and 250 nM Fe(NO_3_)_3_, or solid LB agar plates. Bacterial media was supplemented with ampicillin (100 μg/mL), chloramphenicol (34 μg/mL), kanamycin (50 μg/mL), tetracycline (10 μg/mL) or spectinomycin (50 μg/mL) where appropriate.

### Construction of EHEC deletion strains

Chromosomal deletions of *hha* and *mazEFG* were constructed using the pTOF25 allelic exchange system [60]. Deletion fragments were generated by SOE-PCR and cloned into pTOF25 carrying an FRT–tetRA–FRT cassette for selection. Recombinants were isolated following allelic exchange, confirmed by PCR and sequencing, and the resistance cassette was excised using FLP recombinase (S1 Methods). For complementation, both *hha* and *mazEFG* with their native regulatory sequences were cloned into the medium-copy plasmid pBR322 and verified by sequencing. Overexpression of *mazEFG* was achieved using the aTc-inducible vector pZA21_MCS.

### CRISPRi knockdown of sRNA HnrS

The pCRISPathBrick (pCPB) vector (Addgene, USA) was used for targeted knockdown of *hnrS* in *E. coli* Sakai. sgRNAs targeting *hnrS* were designed, phosphorylated, and annealed. The pCPB vector and sgRNA inserts were digested with BsaI and ligated to generate pCPB::HnrS. Positive clones were confirmed by Sanger sequencing. The verified plasmid was electroporated into *E. coli* Sakai stx(-) and selected on LB agar with kanamycin and chloramphenicol.

### Construction of *hnrS* deletion in EHEC str. Sakai using CRISPR-Cas9

The *hnrS* gene in *E. coli* O157:H7 Sakai stx ⁺ was deleted using a CRISPR-Cas9 system following a two-plasmid approach [61]. A single guide RNA (sgRNA) targeting *hnrS* was cloned into pTarget-F and verified by Sanger sequencing. Homology arms (~300 bp upstream and downstream of *hnrS*) were joined via SOE PCR and inserted into the pTarget-F vector to generate pTargetT::*hnrS*. Plasmids were transformed into DH5α for propagation and sequence verification. For genome editing, pCas expressing the λ-Red recombination system was introduced into Sakai stx ⁺ , induced with L-arabinose at mid-exponential phase, and pTargetT::*hnrS* was electroporated. Transformants were selected on LB agar with kanamycin and spectinomycin, and deletion of *hnrS* was confirmed by colony PCR. Positive colonies were cured of the editing plasmid by growth on LB-kanamycin with IPTG at 42°C.

### Complementation of ∆*hnrS*

For complementation, the HnrS sequence was cloned into the pBAD + 1 expression vector using inverse PCR. The amplified HnrS fragment was treated with DpnI, purified, and ligated into the vector overnight at 16°C. The resulting plasmid was first transformed into DH5α cells and then introduced into EHEC Sakai for functional complementation experiments.

### *In silico* prediction of sRNAs targets

Messenger RNA targets that were found to interact with HnrS from the CLASH dataset were selected for further analysis. Prediction of RNA-RNA interaction was done using computational tool IntaRNA [62].

### GFP translational fusions and sRNA expression vectors

Detailed cloning procedures, including primer sequences and amplification conditions, are provided in S1 Methods. In brief, GFP translational fusions were generated following established operon fusion protocols [63]. For operon fusions, DNA fragments encompassing the C-terminal coding region of the upstream gene, the N-terminal coding region of the downstream gene, and the sRNA-mRNA interaction site were amplified and cloned into the pXG30SF vector encoding superfolder GFP under the P_L*tetO-1*_ promoter. Single-gene fusions were amplified from the transcriptional start site through part of the coding sequence and cloned into the pXG10SF vector. All constructs were verified by plasmid sequencing. For HnrS expression, the sequence was cloned into pZE12 using inverse PCR (S1 Table).

HnrS interactions with target mRNAs were investigated using the two-plasmid system [64]. pXG30::*insert* or pXG10::*insert* was co-transformed with either pZE12::*hnrS* or pJV300 (pZE12 encoding a scrambled RNA sequence) into *E. coli* DH5a. Biological triplicates were grown overnight in LB supplemented with chloramphenicol and ampicillin, overnight cultures were diluted into 15 mL of filtered LB broth supplemented with chloramphenicol and ampicillin, and incubated until OD₆₀₀ reached 0.6. The GFP fluorescence of GFP fusion plasmids was assessed using a POLARstar Omega microplate reader. Fluorescence measurements were taken at an emission wavelength of 510 nm, with an excitation wavelength of 485 nm, which is optimal for GFP fluorescence.

### Northern blot

Total RNA was extracted from E. coli O157:H7 str. Sakai using a guanidinium thiocyanate–phenol method [65] (see S1 Methods for details). Five micrograms of RNA were separated on 8% denaturing polyacrylamide gels and transferred to nylon membranes. RNA was immobilised by UV-crosslinking, and specific sRNAs were detected using ^32^P-labelled oligonucleotide probes. Hybridisation and washes were performed under standard conditions, and signals were visualised using a phosphorimager (Typhoon FLA9500). Detailed protocols are provided in the S1 Methods.

### RNA seq analysis for gene expression

Following RNA extraction, samples were treated with RQ1 RNase-Free DNase (Promega, Cat. no.: M6101) in the presence of RNasin Ribonuclease inhibitor (Promega, Cat. no.: N2511) for 30 minutes at 37 °C to remove residual genomic DNA. RNA was then purified and concentrated using the Zymo RNA Clean & Concentrator kit (Zymo, Cat. no.: R1019), and quality and concentration were assessed using a Nanodrop spectrophotometer and an Agilent TapeStation 4150 to determine RINe values.

The prepared RNA samples were sent to NovogeneAIT Genomics, Singapore for sequencing. Paired-end 150 bp sequencing was performed on the NovaSeq platform, which is optimised for prokaryotic transcriptomes using next-generation sequencing (NGS) technology. Sequencing reads were processed with the READemption v2.0.4 pipeline, aligning reads to the *E. coli* O157:H7 Sakai reference genome (accession number NC_002695.2) and quantifying transcript abundance (S2 Table). RNA sequencing datasets are available at NCBI GEO under the accession GSE311113.

### HnrS prevalence analysis in *E. coli* genomes

Genomic data for 3,230 *E. coli* strains were retrieved from NCBI and analysed to assess strain identity, serotype, and pathovar classification. Key housekeeping genes (*recA*, *purA*, *mdh*, *icd*, *gyrB*, *fumC*, *adk*) and virulence/pathotyping markers were identified using K-mer Analysis (KMA) and Shigella/ ST(E)C Finder tools. Boolean logic rules were applied to classify strains into pathovars such as EHEC, EPEC, EAEC, UPEC, and others. The relative coverage of the HnrS was calculated from KMA output by normalising its depth to housekeeping genes, providing an estimate of gene copy number across strains. Detailed methods are provided in S1 Methods.

### Quantification of bacterial motility

Bacterial strains were grown on LB-agar plates and single colonies were subcultured in LB broth to mid-exponential phase. For single-cell analyses, cultures were diluted to approximately 1 × 10^5^ cells/mL and introduced into flow cells for imaging [66]. Bacterial movement was recorded using phase-contrast microscopy (Nikon or EVOS M7000 [Invitrogen]) with a 20× or 40 × objective respectively, capturing 10–20 second time-lapse videos at 20–31 frames per second. Swimming speeds were determined using custom LabVIEW software [67] and speeds <0.5 µm/s were excluded as non-motile. The resulting data were plotted and analysed using GraphPad Prism 8. For epistasis experiments using the ∆*hha* strain, swim speeds <0.5 mm/s were retained and data processed using custom R scripts to preserve non-motile cells and allow quantification of motility with and without HnrS CRISPRi knockdown.

### Type 3 secretion assay and immunoblotting

Overnight LB cultures were diluted 1:100 into MEM–HEPES with 250 nM Fe(NO_3_)_3_ and 0.1% glucose and grown to OD₆₀₀ ≈ 0.8. Cultures were centrifuged, and supernatants were removed from cell pellets. Cell pellets were retained and resuspended in SDS-PAGE loading buffer. Supernatants were filtered (0.45 μm) and precipitated overnight at 4°C with 10% TCA, using recombinant albumin as a co-precipitant [68]. Precipitated supernatants were resuspended in SDS-PAGE buffer. Both precipitated supernantants and whole cell pellets were heated, and separated on 4–20% gradient gels. For total T3S secretion profiles, supernatants fractions were stained with Coomassie and imaged.

For immunoblotting, supernantant and whole cell proteins were transferred to nitrocellulose, blocked with 8% milk in PBS. EspD was detected using mouse anti-EspD and HRP-conjugated secondary antibodies with ECL. For whole cell fractions, monoclonal mouse anti-DnaK was used as a loading control under the same conditions.

### Cell adhesion assay

Coverslips were placed in 24-well plates and coated with 200 μL of collagen overnight at 4°C.

The following day, wells were gently washed with PBS to remove excess collagen and allowed to air-dry for 3–4 hours. Bovine embryonic lung epithelial cells (EBLs) were seeded at ~10^5^ cells/well in 24-well plates and incubated overnight at 37°C with 5% CO_2_. Bacterial strains grown overnight in LB were subcultured 1:10 in MEM–HEPES with 0.1% glucose and 250 nM FeNO_3_ for ~3 h. One hour before infection, EBL media was replaced with MEM–HEPES, and cells were infected at an MOI of ~100. At 3 and 5 h post-infection, wells were washed, and adhered bacteria were recovered by trypsin and quantified by serial dilution plating on LB agar.

For microscopy, collagen-coated coverslips were fixed with 1% paraformaldehyde for 10 min, permeabilised with 0.1% Triton X-100 for 10 min and blocked with 1 mg/mL bovine serum albumin for 1 h at room temperature. Cells were incubated with rabbit anti-O157 primary antibody (1:50) for 1 h, followed by Alexa Fluor 568–conjugated goat anti-rabbit IgG secondary antibody (1:1000) for 1 h in the dark. Actin filaments were labelled with Phalloidin–Alexa Fluor 488 (BioLegend) for 30 min, and nuclei were counterstained with Hoechst 33342 (1:2000) for 5–10 min. Coverslips were mounted in ProLong Antifade and imaged on a Zeiss LSM900 confocal microscope (63 × oil objective) using 488, 568, and 405 nm excitation. Detailed staining procedures are provided in the S1 Methods.

To quantify pedestal formation, adherent bacteria were identified from the Alexa Fluor 568-labelled anti-O157 immunofluorescence channel in Image J software. Actin pedestal formation was defined by localized accumulation of phalloidin-FITC fluorescence beneath or adjacent to adherent bacteria in merged images. Total adherent bacteria and pedestal-positive bacteria were manually counted across three randomly selected fields per condition, and the percentage of bacteria inducing actin polymerisation was calculated. Values represent the mean obtained from the three independent fields.

## Supporting information

S1 Fig HnrS is encoded within prophages in *E. coli* pathotypes. Prophage regions from eight different E. coli pathotypes were aligned based on sequence similarities between their prophage genomes as determined by BLAST. HnrS is indicated in orange and labelled as EcOnc10 in the alignment. Annotations for phage genes and hypothetical proteins are shown in blue and were retrieved from NCBI; however, no annotations are available for the prophage regions of the EPEC strain E1110019. On the left, the pathotype, serotype, and strain identifiers are listed, followed by either the name of the prophage (for strain Sakai) or the region number assigned by PHASTER. For strain Sakai’s prophage Sp9, the regions responsible for phage replication, regulation, lysis, packaging, head, and tail fibre genes are marked.
(TIF)

S2 Fig HnrS interaction with the MazEF toxin-antitoxin system. **A.** *In silico* prediction of HnrS binding to the *mazF* mRNA, showing the predicted interaction site along with neighbouring genes in the *mazEFG* operon. **B&C.** EMSA of HnrS interaction with the *mazF* coding sequence *in vitro*. 50fmol of either *mazF* (panel **B**) or HnrS (panel **C**) were radiolabelled and incubated with the cognate RNA (concentrations indicated above). **D.** Plaquing assay to assess the sensitivity of *E. coli* K12 wild-type (left) and ∆*mazEFG* (right) to a panel of phages, including P1, T4, λ, and V5-like. Serial dilutions of each phage were spot plated onto the respective bacterial strains, and phage plaques were counted to assess bacterial sensitivity.
(TIF)

S3 Fig HnrS does not repress MazEF toxicity in *E. coli.* A. Growth curves of *E. coli* Top10F’ overexpressing HnrS (pZE12::HnrS) and/or MazEFG (pZA21::mazEFG) under different induction conditions. Cultures were grown at 37°C with shaking for 24 hours in a Bioscreen C system. Panels show growth without aTc (no MazEFG induction) and with increasing aTc concentrations (0.1, 1, 10, 100 ng/mL) to induce MazEFG expression. Control plasmid combinations are indicated as described in Materials and Methods. **B.** Fluorescence of a MazEFG-sfGFP translational fusion in *E. coli* DH5α under different expression conditions of HnrS. Error bars represent standard deviations from biological replicates.
(TIF)

S4 Fig GFP fluorescence versus OD600 for LEE promoter fusions. Individual LEE operon promoters (*ler*, *tir*, *sepL)* were fused to GFP and introduced into EHEC Sakai strains carrying either pBR322 (wild type), ∆*hha* with pBR322, or ∆*hha* complemented with pBR322::*hha*. The promoter-less GFP plasmid pAJR70 served as a control. Fluorescence (GFP) and OD600 were recorded every 20 minutes. Data points represent the mean of three biological replicates, each with two technical duplicates; error bars indicate ±1 SD. A representative experiment of at least three independent trials is shown.
(TIF)

S5 Fig HnrS expression and its impact on mRNAs targets identified by CLASH. **A.** Northern blot showing HnrS expression in wild type (WT), ∆*hnrS*, and complemented strains with pulse expression from pBAD + 1::HnrS. RNA was harvested at mid-exponential (OD_600_ = 0.8) and early stationary phase (OD_600_ = 1.8). **B.** Bar graph showing the expression levels of RNase E CLASH targets of HnrS in RNA seq data. Gene expression is quantified by read counts from biological triplicates, based on DESeq data.
(TIF)

S6 Fig Differential gene expression within the Sp/SpLE prophage regions (indicated above plots). Relative gene expression values are shown for ∆*hnrS*/WT (∆*hnrS*, blue circle), ∆*hnrS* pBAD::*hnrS* (∆*hnrS* pBAD::*hnrS*/∆*hnrS*, yellow triangles), and ∆*hha* (∆*hha*/WT, green squares) [38].
(TIF)

S7 Fig Reciprocal differential gene expression within the Sp/SpLE prophage regions of EHEC str. Sakai ∆*hnrS* and after HnrS pulse expression (∆*hnrS* pBAD::*hnrS*). Genes that have reciprocal regulation in the ∆*hnrS* strain and after HnrS pulse expression (reciprocal |log2FC| > 0.5 and FDR<=0.05 in both conditions) are coloured pink, and genes that do not meet both criteria are coloured grey. Sp/SpLE regions are indicated above each plot. Differential gene expression is plotted on separate x- and y-axis for Sp18 the allow data points to be visualised.
(TIF)

S8 Fig The Mu-like prophage region Sp18 has excised in the ∆*hnrS* mutant. Read counts (log10) were plotted as a heatmap for individual replicates of WT, ∆*hnrS*, and the complemented ∆*hnrS* strain. Genes within the Sp18 prophage region plus 5kb of flanking sequence are indicated below. The genes within the Sp18 region are delimited by the bracket.
(TIF)

S9 Fig Gene Ontology (GO) analysis of differentially expressed genes (DEGs) between the ∆*hnrS* mutant and wild type strain. GO analysis under the Biological Process category identified processes significantly enriched (FDR < 0.05) in the wild type strain compared to the ∆*hnrS* mutant.
(TIF)

S10 Fig GFP translation fusion assay of genes involved in nitrate respiration. **A**. Bar graph depicting the expression of genes involved in nitrate and nitrite respiration, showing reduced expression in ∆*hnrS* relative to WT and complemented strains. Gene expression is quantified from normalised DESeq2 read counts of biological triplicates. *narU.1*, *narG.1*, and *narY.1* correspond to *E. coli* O157:H7 Sakai homologues that are not present in *E. coli* K-12 MG1655, although they share nomenclature with *narU*, *narG*, and *narY*. **B.** Bar graphs show fluorescence from translation fusions of NirB, NapF, NirD, NrfA, NarQ, and Hcp measured in the presence of HnrS or the control plasmid pJV300.
(TIF)

S11 Fig Comparative regulation of genes by Hha and HnrS. Scatterplots of differential gene expression in a *∆hha* and *hnrS* deletion or pulse expression strain. Differential expression data is from a previously published microarray experiment [42] **A.** Scatterplot of significantly differentially regulated genes in a ∆*hha* strain. Nitrate and nitrite metabolism genes that are differentially regulated by HnrS are highlighted in blue. The y-axis indicates log_2_(fold change) in the ∆*hha* background and the x-axis indicates log_2_(fold change) in the ∆*hnrS* strain. **B.** Scatterplot of significantly differentially regulated genes in a ∆*hha* strain compared with pulse expression of HnrS for 15 minutes. As for panel A, nitrate and nitrite metabolism genes regulated by HnrS are indicated in blue. The y-axis indicates log_2_(fold change) after HnrS pulse expression.
(TIF)

S1 Table Bacterial strains, plasmids, primers, guide RNAs, probes, and oligonucleotides used in this study. (XLSX)

S2 Table RNA-seq differential expression analysis of the Δ*hnrS* mutant. Differential expression results for comparisons between the Δ*hnrS* mutant and wild type (Δ*hnrS* vs WT) and between the complemented strain and the Δ*hnrS* mutant (pHnrS complement vs Δ*hnrS*). Separate worksheets contain the results for each comparison and include gene annotations, genomic coordinates, normalized expression values, log2 fold change, P values, and Benjamini–Hochberg adjusted P values (FDR).
(XLSX)

S1 Methods Detailed supplementary experimental procedures, including construction of deletion and complementation strains, CRISPRi knockdown, CRISPR-Cas9 genome editing, RNA extraction, Northern blotting, HnrS prevalence analysis, GFP reporter construction, motility assays, type III secretion assays, cell adhesion assays, and Gene Ontology enrichment analysis. (PDF)

S1 Raw Gel Uncropped original images corresponding to Northern blots, TBE-urea gels, Coomassie-stained SDS-PAGE gels, Western blots, electrophoretic mobility shift assays (EMSAs), and plaque assays presented in Figs 1, 4, 5, S2 and S5. (PDF)
