# Supplementary material for: Phage-encoded sRNA counteracts xenogeneic silencing in pathogenic E. coli
Source: PLoS Pathog. 2026 Aug 18;22(8):e1014486. doi: 10.1371/journal.ppat.1014486 (PMC13502914; doi:10.1371/journal.ppat.1014486)
Supplement: Supplementary file 1 [file ppat.1014486.s014.pdf]

SUPPLEMENTARY METHODS

Phage-encoded sRNA counteracts xenogeneic silencing in pathogenic *E. coli*

Pranita Poudyal, Brandon Sy, Daniel G. Mediati, Michael Payne, Vibhuti Nandel, Dougall Norris, Sean McAteer, Asim Ullah, Serena Li, Lawrence Menz, Egi Kardia, Jacob Scadden, Pietro Ridone, Shafagh Waters, Timothy J. Dallman, Mathew Baker, Ruiting Lan[1], David Gally, Jai J. Tree

Identification and analysis of HnrS within prophages

The *hnrS* sequence was identified across multiple pathogenic *E. coli* strains using Nucleotide BLAST searches. Prophage regions were predicted using PHASTER (1), and sequence alignments were performed to assess both the conservation and genomic context of *hnrS*. Representative *hnrS*-encoding prophage regions were visualised using Easyfig, which demonstrated that *hnrS* was consistently located upstream of genes encoding phage packaging and head proteins. Comparative analyses confirmed that *hnrS* is highly conserved within prophage regions and is restricted to prophage-associated elements across all strains examined.

Construction and validation of chromosomal deletions and complementation plasmids

Chromosomal deletions of *hha* and *mazEFG* were generated using the pTOF25 allelic exchange system (2). For each target, approximately 500 nt regions flanking the gene(s) of interest were amplified from genomic DNA using primer pairs designed for splicing by overlap extension (SOE) PCR (Supplementary Table 1).

For *hha*, the upstream flanking region was amplified using primers PP_hha_SmaI_A_R and PP_hha_SOE_B_F, while the downstream region was amplified using PP_hha_SOE_C_R and PP_hha_SalI_D_F. For *mazEFG*, the upstream region was amplified using PP_mazE_SmaI_A_R and PP_mazE_SOE_B_F, and the downstream region using PP_mazF_SOE_C_R and PP_mazF_SalI_D_F.

Primers PP_hha_SOE_B_F and PP_hha_SOE_C_R (for *hha*), and PP_mazE_SOE_B_F and PP_mazF_SOE_C_R (for *mazEFG*), contained overlapping sequences to facilitate fusion of the upstream and downstream fragments during SOE PCR. The resulting contiguous PCR products were gel-purified and cloned into the pTOF25 plasmid using SmaI and SalI restriction sites. Restriction digests were incubated at 37°C for 1 h, with FastAP thermosensitive alkaline phosphatase added after 45 min to reduce vector self-ligation, followed by enzyme inactivation at 65°C for 20 min. Ligation reactions were performed using T4 DNA ligase at room temperature for 1 h and transformed into *E. coli* DH5α cells. All constructs were verified by Sanger sequencing.

A tetracycline resistance cassette (FRT–tetRA–FRT) was excised from pTOF1 using NotI and inserted into the pTOF25 allelic exchange plasmids. Transformants were selected on LB agar containing chloramphenicol (20 µg/mL) and tetracycline (10 µg/mL) at 30°C. Allelic exchange was induced by serial passage on LB agar plates containing 6% sucrose without NaCl at 42°C. Recombinant colonies were screened for tetracycline resistance and chloramphenicol sensitivity, and deletion of the target genes (*hha* or *mazEFG*) was confirmed by colony PCR.

For both *hha* and *mazEFG* deletion strains, the tetRA cassette was subsequently excised by electroporation of pCP20, which encodes FLP recombinase. Correct deletion of each target locus was confirmed by PCR and Sanger sequencing.

Knock-down of HnrS using CRISPRi in EHEC

Targeted knockdown of *hnrS* was performed in *E. coli* Sakai stx(-) using the CRISPathBrick CRISPR interference (CRISPRi) system (10). The pCRISPathBrick (pCPB) plasmid, obtained from Addgene (USA), encodes catalytically inactive dCas9 and enables insertion of spacer-repeat bricks (SRBs) containing single guide RNAs (sgRNAs).

SRB oligonucleotides containing the *hnrS*-targeting sgRNA sequence (Supplementary Table 1) were synthesised with 5′ phosphorylation (IDTDNA). Complementary oligonucleotides were annealed by combining equimolar concentrations (50 µM total) in IDTDNA duplex buffer (100 mM potassium acetate, 30 mM HEPES, pH 7.5). Successful annealing was confirmed by gel electrophoresis.

The pCPB vector (500 ng) and annealed oligonucleotides (200 ng) were digested with BsaI. FastAP thermosensitive alkaline phosphatase was added to reduce vector self-ligation, followed by BsaI inactivation. Ligation reactions containing 40 ng pCPB vector and 80 ng annealed oligonucleotide insert were incubated at room temperature (21°C) for 1.5 h, generating the pCPB::*hnrS* construct.

Ligation products were transformed into competent *E. coli* DH5α cells. Positive transformants were screened and verified by Sanger sequencing. A glycerol stock was prepared from a sequence-confirmed clone for long-term storage. The verified pCPB::*hnrS* plasmid was subsequently electroporated into EHEC stx(-) cells, and transformants were selected on LB agar supplemented with kanamycin and chloramphenicol.

Construction of HnrS deletion in EHEC str. Sakai using CRISPR-Cas9

CRISPR-Cas9-mediated deletion of *hnrS* in *E. coli* O157:H7 Sakai stx+ was performed using the two-plasmid system described previously (7). The sgRNA targeting *hnrS* was introduced into pTarget-F by inverse PCR using Phusion polymerase with primers BS_EcOnc10_sgRNA_F and BS_pTargetF_R_5P (5′ phosphorylated) (Supplementary Table 1). Following amplification, 1 µL DpnI was added and incubated at 37°C for 1 h. The linearised vector was purified using the Wizard® SV Gel and PCR Clean-Up System (Promega, Catalogue No. A9282), ligated using T4 DNA ligase (ThermoFisher), and transformed into competent *E. coli* DH5α cells by heat shock transformation. Plasmids were extracted and correct insertion of the *hnrS* sgRNA into pTargetF was confirmed by Sanger sequencing using the BS_pTarget_F screening primer. The resulting construct was termed pTargetF::*hnrS*.

Approximately 300 nt homology arms upstream and downstream of *hnrS* were amplified from *E. coli* O157:H7 Sakai stx+ genomic DNA using primer pairs BS_EcOnc10_Down_F/BS_EcOnc10_Down_R and BS_EcOnc10_UP_F/BS_EcOnc10_UP_R (Supplementary Table 1). The two homology arms were fused by splicing by overlap extension (SOE) PCR using primers BS_EcOnc10_Down_R and BS_EcOnc10_UP_F to generate a repair template lacking *hnrS*.

The plasmid pTargetF::*hnrS* and the SOE PCR repair template were digested with HindIII-HF and XhoI (New England Biolabs, Catalogue Nos. R3104S and R0146S). To reduce vector self-ligation, 1 U FastAP thermosensitive alkaline phosphatase (ThermoFisher Scientific, Catalogue No. EF0651) was added to the pTargetF::*hnrS* digestion reaction after 45 min. Reactions were heat-inactivated at 65°C for 20 min. Ligation reactions (20 µL total volume) containing 1× T4 DNA ligase buffer and 1 U T4 DNA ligase (ThermoFisher Scientific, Catalogue No. EL0011) were prepared using a 1:3 vector-to-insert molar ratio and incubated at 20°C for 1.5 h. The resulting construct containing both the sgRNA and homologous recombination template was termed pTargetT::*hnrS*.

The pTargetT::*hnrS* plasmid was transformed into competent *E. coli* DH5α cells by heat shock transformation. Transformants were selected on LB agar supplemented with spectinomycin and screened for insertion of the homology arms using primers BS_EcOnc10_Down_F and BS_EcOnc10_UP_R. Correct insertion was confirmed by Sanger sequencing using primer BS_EcOnc10_Down_F.

All incubations were performed at 30°C unless otherwise stated. The pCas plasmid was electroporated into *E. coli*O157:H7 Sakai stx+ cells, and transformants were streaked for single colonies on LB-Km50 agar plates. Single colonies were grown overnight in LB-Km50 broth and subcultured 1:100 the following day. When cultures reached an OD600 of 0.45–0.6, λ-Red recombination was induced with 50 mM L-arabinose for 30 min. Cells were harvested and electroporated with pTargetT::*hnrS* as described above. Transformants were selected on LB-Km50Sm50 agar plates. Colony PCR was used to verify the desired chromosomal deletion, and plasmids were cured by streaking positive colonies onto LB-Km50 agar supplemented with 0.5 mM IPTG followed by overnight incubation at 42°C.

RNA extraction

RNA was extracted using the guanidinium thiocyanate–phenol method (3) with minor modifications. Bacterial cultures were grown in LB broth, minimal M9 medium, or MEM-HEPES supplemented with 0.1% glucose and 250 nM $Fe(NO_3)_3$ to the desired growth phase as determined by OD600. Cells were harvested by centrifugation at 4000 × g for 10 min at 4°C and resuspended in 1 mL of a 1:1 mixture of GTC buffer and phenol together with 250 µL of 0.1 mm zirconia-silica beads (Biospec Products, Catalogue No. 11079101z). GTC buffer consisted of 4 M guanidinium thiocyanate, 2% Sarkosyl, 50 mM Tris-HCl (pH 8.0), 10 mM EDTA, and 1% β-mercaptoethanol. Cells were lysed by vortexing followed by incubation at 65°C for 5 min.

Lysates were mixed with 350 µL chloroform and 120 µL 3 M sodium acetate (pH 5.2), vortexed for 20 s, and centrifuged at maximum speed for 5 min at room temperature. The aqueous phase was sequentially extracted with phenol:chloroform:isoamyl alcohol (25:24:1) followed by chloroform alone. RNA was precipitated from the final aqueous phase using 2.5 volumes of 100% ethanol and 15 µg/mL GlycoBlue coprecipitant (ThermoFisher, Catalogue No. AM9515) at −80°C overnight. Samples were centrifuged at 16,000 × g for 30 min at 4°C, washed twice with 70% ethanol, air dried, and resuspended in 40 µL nuclease-free water.

Northern blotting

RNA samples were separated on 8% denaturing polyacrylamide gels prepared in TBE-urea buffer (1× TBE, 8 M urea, 8% acrylamide). Gels were polymerised using TEMED and 1%

ammonium persulfate (APS) and pre-run at 200 V for 30 min before sample loading. RNA samples were mixed with 2× formamide loading buffer (80% formamide, 10 mM EDTA, 0.025% bromophenol blue, 0.025% xylene cyanol), denatured at 65°C for 10 min, and electrophoresed at 200 V for at least 3 h.
Separated RNA was transferred to a nylon membrane using a CBS Scientific Large Blotting System (Catalogue No. EBU-102) at 30 V in 0.5× TBE buffer overnight. RNA was UV-crosslinked to the membrane using a Stratagene Auto-Crosslinker at 1200 mJ and prehybridised in Ambion™ ULTRAhyb™ Ultrasensitive Hybridization Buffer (Catalogue No. AM8670) at 30°C for 30 min.

Oligonucleotide probes (30–35 nt) were designed using NetPrimer and Integrated Genome Browser and radiolabelled with ^32P-ATP using T4 polynucleotide kinase (PNK). Labelling reactions contained 1.5 µL 10 mM oligonucleotide probe, 2.5 µL ^32P-ATP, 1.5 µL 10× PNK buffer, 1 µL T4 PNK, and 9 µL MilliQ water and were incubated at 37°C for 1 h. Radiolabelled probes were purified using GE Healthcare Illustra Microspin™ G-50 columns (Catalogue No. 27-53301) according to the manufacturer's instructions.

Purified BS_HnrS_NB probes (Table 3.4) were hybridised to membranes overnight at 30°C in a humidified chamber. Membranes were washed three times in 2× SSPE buffer (0.3 M NaCl, 20 mM NaH_2PO_4, 2 mM EDTA) containing 0.1% SDS for 10 min at 42°C before exposure to a BAS Storage Phosphor Screen. Signals were detected using a Typhoon™ FLA9500 imaging system.

HnrS prevalence in *E. coli* genomes

Genomic data for 3,230 E. coli strains were retrieved from the NCBI database and processed using a series of bioinformatics tools to identify key genetic markers and assess the strains' pathogenic potential. Initially, the data underwent K-mer Analysis (KMA) to detect essential housekeeping genes such as *recA*, *purA, mdh, icd, gyrB, fumC,* and *adk,* which were used to confirm strain identity and estimate genome depth. In addition, KMA identified pathotyping genes like *eae, escV, stx1,* and *stx2,* allowing for the classification of strains into distinct E. coli pathovars, including EHEC, EPEC, EAEC, and others. A detailed list of these pathotyping genes, along with their associated pathovars, is provided in Table A

To determine the copy number of *hnrS*, a relative coverage approach was used based on the KMA results. First, the sequencing reads for each strain were processed using the test_commandPP script (Section 3.3.5), which runs KMA along with ShigeFinder and STECFinder to identify genes present in the sample. During this process, the KMA output (*_kma_out.res) was analysed to identify the coverage depth of the *hnrS* gene among other genes. The *hnrS* gene's coverage was extracted from the KMA results, and the relative coverage was calculated by comparing the observed coverage of HnrS to the overall genomic depth of the strain, using housekeeping genes for normalisation. This relative coverage of *hnrS* was then used as an indicator of its copy number, with higher relative coverage suggesting a greater number of copies of the *hnrS* gene present in the strain. This calculation was done in the post-processing step through the Python script process_ecolitypingv3, which interpreted the KMA results and calculated the relative coverage for each strain.

Shigella Finder was used to categorise strains as Shigella, Enteroinvasive E. coli (EIEC), or non-Shiga toxigenic E. coli. Based on specific genetic markers, strains were further classified into "Shigella," "EIEC," or "Shigella/EIEC Unclustered," with additional sub-classifications

based on serotype. Meanwhile, STEC Finder was used to identify serotypes within the STEC group by detecting serotype-specific markers such as *stx1*, *stx2*, *eae*, and *escV*. This enabled precise classification of strains into known serotypes, including O157:H7, and facilitated grouping them into categories like EHEC, based on their genetic profile. The STEC Finder software relies on a predefined database of serotype-specific markers to assign each strain to its corresponding serotype.

Table A. Pathotying genes with associated pathovars.

S.N	Gene	Pathovar	Reference
**1.**	*fyuA*	UPEC	(4)
**2.**	*papC*	UPEC	(4)
**3.**	*hlyA*	UPEC	(4)
**4.**	*traT*	UPEC	(4)
**5.**	*escV*	LEE positive strains (EPEC, ATEC, STEC)	(5)
**6.**	*bfp*	EPEC, atypical, Samonella	(5)
**7.**	*stx1*	STEC	(5)
**8.**	*stx2*	STEC	(5)
**9.**	*aaic*	EAEC	(5)
**10.**	*aggR*	EAEC	(5)
**11.**	*invA*	EIEC	(5)
**12.**	*uidA*	E.coli	(5)
**13.**	*eae*	EPEC and EHEC	(5)
**14.**	*ipaH*	EIEC and Shigella	(6)
**15.**	*daaD*	DAEC	(5)
**16.**	*virG*	EIEC and Shigella	(6)
**17.**	*est*	ETEC	(5)
**18.**	*elt*	ETEC	(5)

To further refine pathovar classification, a Boolean logic expressions were applied, as detailed in Table B. This algorithm enabled classification of strains into specific pathovars based on the presence or absence of key genes. For instance, EHEC strains were identified by the presence of *eae* or *escV* along with *stx1* or *stx2* but without *bfp*, while Uropathogenic Escherichia coli (UPEC) strains were defined by multiple virulence factors such as *fyuA*, *traT*, *hlyA*, and *papC*.

Finally, the relative coverage of the HnrS gene was assessed by comparing its coverage to the overall genome depth, providing a measure of its prevalence across different E. coli strains. The final output compiled essential information for each strain, including strain ID, Shigella/EIEC status (from Shigella Finder), serotype (from STEC Finder), gene-based pathotype (from KMA), and the list of detected pathotyping genes. This dataset was used to assess the occurrence of HnrS across various genomes and strains, aiding in the prediction of its presence in virulent strains.

Codes for performing HnrS prevalence analysis in *E. coli* genomes is in https://github.com/pranitapoudyal/EcOnc10-prevalence-in-E.-coli-genomes-with-M.-Payne

Table B. Boolean logic expression to classify pathovars

Pathovar	*Boolean Logic Expression*
EHEC	((*eae* OR *escV*) AND *stx*) NOT *bfp*
EPEC	(*eae* OR *escV)* NOT *stx*
EAEC	(*aggR* AND *aaiC*) NOT *stx*
DAEC	(*daaD* OR *afaD*) NOT (*stx* OR *bfp*)
UPEC	((*fyuA* AND *traT*) OR (*fyuA* AND *hlyA*) OR (*fyuA* AND *papC*) OR (*traT* AND *hlyA*) OR (*traT* AND *papC*) OR (*hlyA* AND *papC*))
ETEC	(*elt* OR *est*)
EIEC	(*invA* AND *ipaH*) AND NOT (*stx* OR *bfp*)
Shigella	(*ipaH* and *virG*)
Salmonella	(*invA*)

Pulse expression of HnrS for RNA-sequencing

The sequence for *hnrS* was cloned into the pBAD+1 vector by inverse PCR using primers pBAB_PP_EcOnc10_F and pBAD_PP_5P_R (Supplementary Table 1). The resulting construct contained 66 nucleotides of *hnrS*. PCR amplification was performed using Phusion polymerase, followed by digestion with 1 µL DpnI (NEB) at 37°C for 1 h to remove template plasmid DNA. Amplicons were purified using the Wizard® SV Gel and PCR Clean-Up System (Promega), ligated overnight at 16°C using T4 DNA ligase, and transformed into competent *E. coli* DH5α cells. Verified plasmids were subsequently transformed into EHEC str. Sakai Δ*hnrS*.

Construction of GFP translational fusions and sRNA expression vectors

GFP translational fusions were constructed following the operon fusion protocol described by Menz (11) and based on the approach of Urban and Vogel (12). Primers were designed to amplify a fragment from the polycistronic *mazEFG* locus for translational fusion with GFP. The forward primer annealed to the C-terminal coding region of the upstream *mazF* gene, including the predicted interaction site (seed sequence), spanning 87 bp encoding the final 29 amino acids of MazF. An NsiI restriction site extension (ATGCAT) was incorporated at the 5′ end to maintain the correct reading frame with the upstream coding sequence.

The reverse primer annealed to the N-terminal coding region of the downstream *mazG* gene, spanning 84 bp encoding the first 27 amino acids of MazG. An NheI restriction site extension (GCTAGC), corresponding to the second and third codons of GFP, was included to maintain the correct translational reading frame with the downstream coding region. Primer design also ensured the absence of internal NsiI or NheI restriction sites within the amplified fragment.
The resulting PCR product was cloned into the pXG30SF vector using NheI and NsiI (FastDigest, ThermoFisher). The pXG30SF vector encodes superfolder GFP under control of the *PLtetO-1* promoter.

For *hnrS* expression constructs, *hnrS* was cloned into pZE12 by inverse PCR using Phusion polymerase with primers pZE12-inverse PCR.F and pZE12-inverse PCR.5P.R (5′ phosphorylated) (Supplementary Table 1), where pZE12-inverse PCR.F contained the *hnrS* sequence. Following amplification, products were purified using the Wizard® SV Gel and PCR Clean-Up System (Promega). Purified amplicons were ligated using T4 DNA ligase (ThermoFisher) to recircularise the vector and incubated at room temperature for 1 h before heat shock transformation into ultra-competent *E. coli* DH5α cells. Colonies were screened

using primers pZE12.seq.F and pZE12.seq.R, and clones containing inserts of the expected size were selected. Plasmids were extracted using the Wizard® Plus SV Minipreps DNA Purification System (Promega) according to the manufacturer's instructions, and correct insertion of *hnrS* was confirmed by Sanger sequencing using the forward screening primer (Supplementary Table 1).

Quantification of bacterial motility

For plate-based motility assays, bacterial strains were streaked onto LB agar plates and incubated overnight at 37°C. Single colonies were inoculated into LB broth containing ampicillin (100 µg/mL) and grown to an OD600 of 0.6. Aliquots (0.2 µL) were inoculated onto LB plates containing 0.2% agar and incubated at 30°C. Motility was assessed by measuring the diameter of the resulting bacterial flare.

For single-cell motility assays, overnight cultures were subcultured 1:100 into Tryptone Broth (1% [w/v] tryptone, 0.5% [w/v] NaCl) to a starting OD600 of 0.1 and grown to late exponential phase. Cultures were diluted to approximately 1×10^5 cells/mL prior to imaging. Diluted bacterial suspensions were introduced into flow cells consisting of microscope slides and coverslips separated by tape to generate a confined channel for bacterial movement (13). Samples were equilibrated on the microscope stage for 10 min prior to imaging.

Motility was visualised using either a Nikon phase-contrast microscope equipped with a 40× objective lens or EVOS™ M7000 (Invitrogen) using a 20x objective. Time-lapse videos were acquired using a Chameleon3 CM3 camera (Point Grey Research) over 10-20 s at 20-31 frames per second, generating 324-400 frames per recording. Swimming speeds were quantified using custom LabVIEW 2023 software (14) and analysed using GraphPad Prism 8. For Figure 4D, because the Δ*hha* strain is largely non-motile, swim speeds were calculated from X–Y coordinates (µm) as a function of time using custom R scripts to retain non-motile cells that are removed during filtering in LabView software.

Type 3 secretion profiles and western blotting

Type III secretion (T3S) assays were performed as previously described (15). Overnight LB cultures were diluted 1:100 into MEM-HEPES supplemented with 250 nM $Fe(NO_3)_3$ and 0.1% glucose and grown to an OD600 of 0.8. Culture supernatants were collected by centrifugation at 4000 rpm for 30 min and filtered through 0.45 µm low protein-binding syringe filters (Millex-HP Syringe Filter Unit). Secreted proteins were precipitated overnight at 4°C using 10% trichloroacetic acid (TCA; Sigma-Aldrich) with 100 µg recombinant albumin (rAlbumin; New England BioLabs, Catalogue No. B9200S) added as a co-precipitant and loading control. Precipitated proteins were collected by centrifugation at 4000 × g for 30 min at 4°C. Pellets were air dried, resuspended in 150 µL loading dye, heated at 95°C for 10 min, and separated by SDS-PAGE using 4–20% Mini-PROTEAN TGX Precast Gels (Bio-Rad, Catalogue No. 4561093). Gels were stained with Coomassie and imaged using a Bio-Rad ChemiDoc system with default white-light illumination settings.

Proteins separated by SDS-PAGE were transferred onto Protran nitrocellulose membranes (Cytiva Amersham, Catalogue No. 15249794) using a Trans-Blot electrophoretic transfer cell (Bio-Rad). Membranes were blocked in 8% (w/v) skim milk powder prepared in phosphate-buffered saline (PBS) for 1 h at room temperature and washed three times with PBS.

EspD proteins were detected by immunoblotting using mouse monoclonal anti-EspD primary antibodies (kindly provided by Professor T. Chakraborty, University of Giessen) and rabbit polyclonal anti-mouse IgG HRP-conjugated secondary antibodies (Cell Signaling Technology, Catalogue No. 7076), both diluted 1:2000. Membranes were incubated with primary antibodies overnight at 4°C on a platform shaker, washed three times for 10 min in PBS, and subsequently incubated with secondary antibodies for 1 h at 4°C on a platform shaker followed by three additional 10 min PBS washes.

For enhanced chemiluminescence (ECL) detection, membranes were incubated for 5 min at room temperature in a 1:1 mixture of ECL Solution 1 and ECL Solution 2 (Amersham). Chemiluminescent signals were detected using a ChemiLITE Chemiluminescence Imaging System (Cleaver Scientific).

As a loading control, mouse monoclonal anti-DnaK antibody (Roslin Institute, University of Edinburgh) was used to detect DnaK in whole-cell lysates prepared from the corresponding bacterial cultures. Immunoblotting was performed as described above using the same antibody dilutions and secondary antibody conditions**.**

LEE1, LEE4 and LEE5 translation GFP reporter assays.

Plasmid-based LEE1, LEE4, and LEE5 promoter and 5′ untranslated region (UTR) translational fusions to green fluorescent protein (GFP) (16). The constructs consisted of the native promoters and corresponding coding regions of key genes within each operon — *ler* for LEE1, *sepL* for LEE4, and *tir* for LEE5 — cloned into the pAJR70 vector to generate translational GFP fusions.
GFP reporter plasmids were introduced into EHEC str. Sakai pBR322 (wild type), EHEC Sakai Δ*hha* pBR322, and EHEC str. Sakai Δ*hha* pBR322::*hha* strains by electroporation (Section 2.2.7). Transformants were selected on LB agar supplemented with ampicillin (100 μg/mL) and chloramphenicol (50 μg/mL).

Cultures were grown in MEM-HEPES supplemented with chloramphenicol, ampicillin, glucose, and iron under conditions promoting LEE expression. Bacterial growth (OD600) and GFP fluorescence were measured hourly in black 96-well FluoroNunc plates using a FLUOstar Optima fluorimeter. Fluorescence values were normalised to bacterial growth and plotted against OD600 using GraphPad Prism software. The promoterless pAJR70 plasmid was included as a negative control to determine background fluorescence arising from the bacterial strain and growth medium.

Cell Adhesion Assays

Coverslips were placed in 24-well plates and coated with 200 µL of collagen overnight at 4°C. The following day, wells were gently washed with PBS to remove excess collagen and allowed to air-dry for 3–4 hours. Bovine epithelial cells (EBLs) were seeded at approximately 1×10^5 cells per well and incubated overnight at 37°C with 5% CO_2 to allow adherence. Overnight bacterial cultures were sub-cultured 1:10 in MEM supplemented with 0.1% glucose and 250 nM $Fe(NO_3)_3$ and incubated for approximately 3 hours. One hour prior to infection, the EBL growth medium was replaced with MEM to match the bacterial growth medium, and cells were then infected with 100 µL of bacterial culture to achieve a multiplicity of infection (MOI) of ~100. Infected cultures were incubated for 3 hours at 37°C, after which non-adherent bacteria were removed by two washes with MEM. Following an additional 2-hour incubation, wells

were washed twice with phosphate-buffered saline (PBS), and cells were fixed with 100 µL of 1% paraformaldehyde (PFA) per well for 10 minutes at room temperature. Fixed cells were washed with PBS and stored at 4°C until immunostaining.

For immunostaining, fixed cells were permeabilized with 200 µL of 0.1% Triton X-100 per well for 10 minutes at room temperature and washed twice with PBS. Cells were blocked with 200 µL of 1 mg/mL bovine serum albumin (BSA) in PBS for 1 hour at room temperature and washed twice with PBS. Primary staining was performed using 200 µL of rabbit anti-O157 antibody (1:50 dilution) for 1 hour at room temperature, followed by three 5-minute washes with PBS. Secondary staining was carried out with 200 µL of goat anti-rabbit IgG Alexa Fluor 568 (1:1000 dilution) for 1 hour at room temperature in the dark, followed by three 5-minute washes with PBS. Actin filaments were stained using 200 µL of Phalloidin Alexa Fluor 488 (BioLegend) for 30 minutes at room temperature in the dark, followed by two washes with PBS, and nuclei were stained with 100 µL of Hoechst 33342 (1:2000 dilution) for 5–10 minutes at room temperature in the dark, followed by three washes with PBS.

Coverslips were then carefully removed and inverted onto a drop of ProLong antifade mounting medium on glass slides and allowed to cure for 24 hours. Imaging was performed at at the Katherina Gaus Centre for Microscopy (UNSW) using a Zeiss LSM900 inverted confocal laser scanning microscope with a 63× oil immersion objective. Fluorescence was detected using 488 nm excitation for Phalloidin, 405 nm for Hoechst, and 633 nm for Alexa Fluor 568, and images were processed and merged using ImageJ software.

Gene Ontology (GO) enrichment analysis

Gene Ontology (GO) enrichment analysis was performed to investigate the functional categories associated with genes differentially expressed between the *hnrS* deletion mutant and the wild-type strain. Differentially expressed genes identified by DESeq2 with an absolute log2 fold change ≥ 1 were included in the analysis. GO enrichment analysis was conducted in R using the clusterProfiler package across the three GO ontology categories: biological process, molecular function, and cellular component. Enriched GO terms were visualised using ggplot2-generated scatter plots, where point size represented the number of genes associated with each GO term and colour indicated the adjusted *P*-value.

REFERENCES

1. Arndt D, Grant JR, Marcu A, Sajed T, Pon A, Liang Y, Wishart DS. 2016. PHASTER: a better, faster version of the PHAST phage search tool. Nucleic Acids Res 44:W16-21.
2. Merlin C, McAteer S, Masters M. 2002. Tools for characterization of Escherichia coli genes of unknown function. J Bacteriol 184:4573-81.
3. Tollervey D, Mattaj IW. 1987. Fungal small nuclear ribonucleoproteins share properties with plant and vertebrate U-snRNPs. Embo j 6:469-76.
4. Kudinha T, Kong F, Johnson JR, Andrew SD, Anderson P, Gilbert GL. 2012. Multiplex PCR-Based Reverse Line Blot Assay for Simultaneous Detection of 22 Virulence Genes in Uropathogenic Escherichia coli. Applied and Environmental Microbiology 78:1198-1202.
5. Müller D, Greune L, Heusipp G, Karch H, Fruth A, Tschäpe H, Schmidt MA. 2007. Identification of unconventional intestinal pathogenic Escherichia coli

isolates expressing intermediate virulence factor profiles by using a novel single-step multiplex PCR. Appl Environ Microbiol 73:3380-90.
6. Vidal M, Kruger E, Durán C, Lagos R, Levine M, Prado V, Toro C, Vidal R. 2005. Single multiplex PCR assay to identify simultaneously the six categories of diarrheagenic Escherichia coli associated with enteric infections. J Clin Microbiol 43:5362-5.
7. Jiang Y, Chen B, Duan C, Sun B, Yang J, Yang S. 2015. Multigene editing in the Escherichia coli genome via the CRISPR-Cas9 system. Appl Environ Microbiol 81:2506-14.
8. Islam MR, Ogura Y, Asadulghani M, Ooka T, Murase K, Gotoh Y, Hayashi T. 2012. A sensitive and simple plaque formation method for the Stx2 phage of Escherichia coli O157:H7, which does not form plaques in the standard plating procedure. Plasmid 67:227-35.
9. Bonanno L, Loukiadis E, Mariani-Kurkdjian P, Oswald E, Garnier L, Michel V, Auvray F, Griffiths MW. 2015. Diversity of Shiga Toxin-Producing Escherichia coli (STEC) O26:H11 Strains Examined via <i>stx</i> Subtypes and Insertion Sites of Stx and EspK Bacteriophages. Applied and Environmental Microbiology 81:3712-3721.
10. Cress BF, Toparlak Ö D, Guleria S, Lebovich M, Stieglitz JT, Englaender JA, Jones JA, Linhardt RJ, Koffas MA. 2015. CRISPathBrick: Modular Combinatorial Assembly of Type II-A CRISPR Arrays for dCas9-Mediated Multiplex Transcriptional Repression in E. coli. ACS Synth Biol 4:987-1000.
11. Menz L. 2017. The role of the small RNA ecOnc10 in regulating MazEF in enterohaemorrhagic Escherichia coli O157:H7. Bachelor of Biotechnology. UNSW, Sydney.
12. Urban JH, Vogel J. 2007. Translational control and target recognition by Escherichia coli small RNAs in vivo. Nucleic Acids Res 35:1018-37.
13. Palma V, Gutiérrez MS, Vargas O, Parthasarathy R, Navarrete P. 2022. Methods to Evaluate Bacterial Motility and Its Role in Bacterial-Host Interactions. Microorganisms 10.
14. Ishida T, Ito R, Clark J, Matzke NJ, Sowa Y, Baker MAB. 2019. Sodium-powered stators of the bacterial flagellar motor can generate torque in the presence of phenamil with mutations near the peptidoglycan-binding region. Mol Microbiol 111:1689-1699.
15. Roe AJ, Yull H, Naylor SW, Woodward MJ, Smith DG, Gally DL. 2003. Heterogeneous surface expression of EspA translocon filaments by Escherichia coli O157:H7 is controlled at the posttranscriptional level. Infect Immun 71:5900-9.
16. Fernandez-Brando RJ, Yamaguchi N, Tahoun A, McAteer SP, Gillespie T, Wang D, Argyle SA, Palermo MS, Gally DL. 2016. Type III Secretion-Dependent Sensitivity of Escherichia coli O157 to Specific Ketolides. Antimicrob Agents Chemother 60:459-70.
