# Supplementary material for: Phage-encoded sRNA counteracts xenogeneic silencing in pathogenic E. coli
Source: PLoS Pathog. 2026 Aug 18;22(8):e1014486. doi: 10.1371/journal.ppat.1014486 (PMC13502914; doi:10.1371/journal.ppat.1014486)

Figure 1B. Northern blot analysis of HnrS expression in LB, M9 minimal medium, and MEM-HEPES at different growth phases of EHEC str. Sakai *Δstx1 stx2A::kan* pCPB.

Samples were loaded from left to right as follows:

Lane 1: Ladder (Not Visible in Northern)
Lane 2: Mid-exponential phase (OD600 = 0.6)
Lane 3: Late exponential phase (OD600 = 1.0)
Lane 4: Early stationary phase (OD600 = 1.8)

Lanes 5–7: M9 minimal medium
Lane 5: Mid-exponential phase (OD600 = 0.6)
Lane 6: Late exponential phase (OD600 = 1.0)
Lane 7: Early stationary phase (OD600 = 1.8)

Lanes 8–10: MEM-HEPES
Lane 8: Mid-exponential phase (OD600 = 0.6)
Lane 9: Late exponential phase (OD600 = 1.0)
Lane 10: Early stationary phase (OD600 = 1.8)

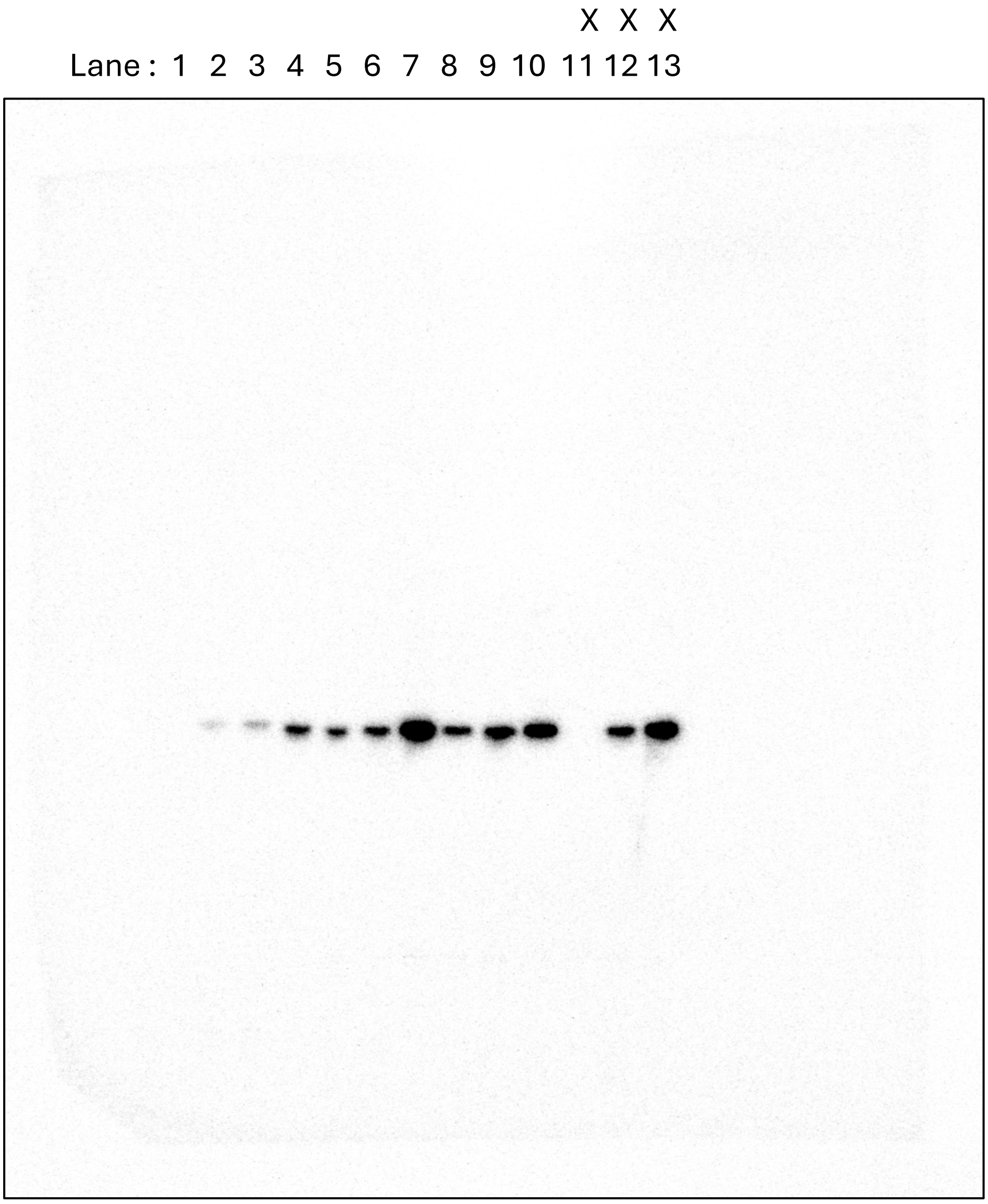

Figure 1B. TBE Urea gel for expression of 5s rRNA in LB, M9 minimal medium, and MEM-HEPES at different growth phases.

Samples were loaded from left to right as follows:
Lane 1: Ladder (Low Range ssRNA Ladder- NEB)
Lanes 2–4: LB medium
Lane 2: Mid-exponential phase (OD600 = 0.6)
Lane 3: Late exponential phase (OD600 = 1.0)
Lane 4: Early stationary phase (OD600 = 1.8)

Lanes 5–7: M9 minimal medium
Lane 5: Mid-exponential phase (OD600 = 0.6)
Lane 6: Late exponential phase (OD600 = 1.0)
Lane 7: Early stationary phase (OD600 = 1.8)

Lanes 8-10: MEM-HEPES
Lane 8: Mid-exponential phase (OD600 = 0.6)
Lane 9: Late exponential phase (OD600 = 1.0)
Lane 10: Early stationary phase (OD600 = 1.8)

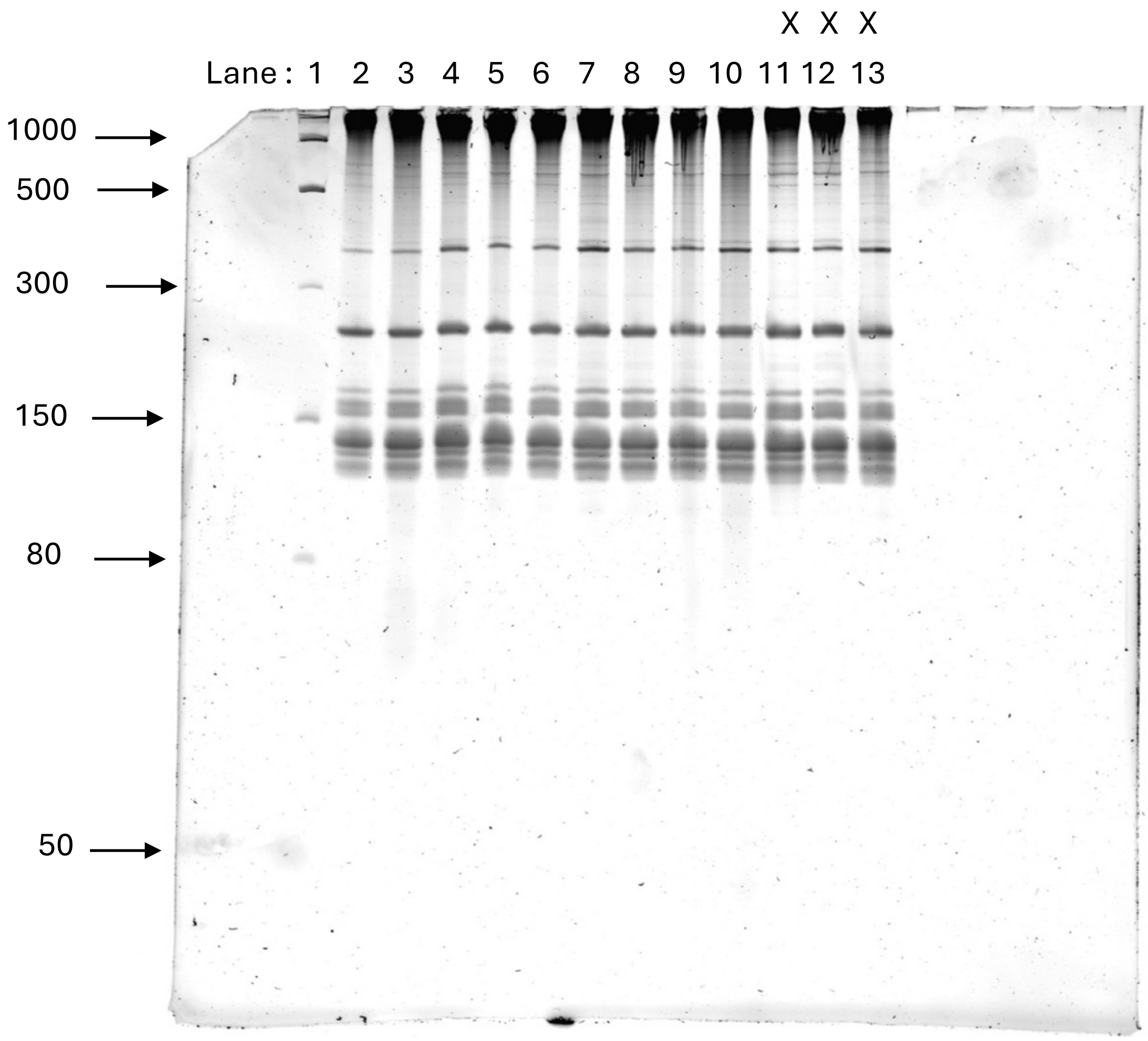

Figure 4C. Northern blot analysis of HnrS expression in wildtype and HnrS CRISPRi knockdown strains (pCPB vector) in MEM - HEPES

Samples were loaded from left to right as follows:
Lane 1: Ladder (Not Visible in Northern)
Lane 10: EHEC str. Sakai *Δstx1 stx2A::kan* pCPB (wildtype)
Lane 11: EHEC str. Sakai *Δstx1 stx2A::kan* pCPB::sgHnrS (knockdown)

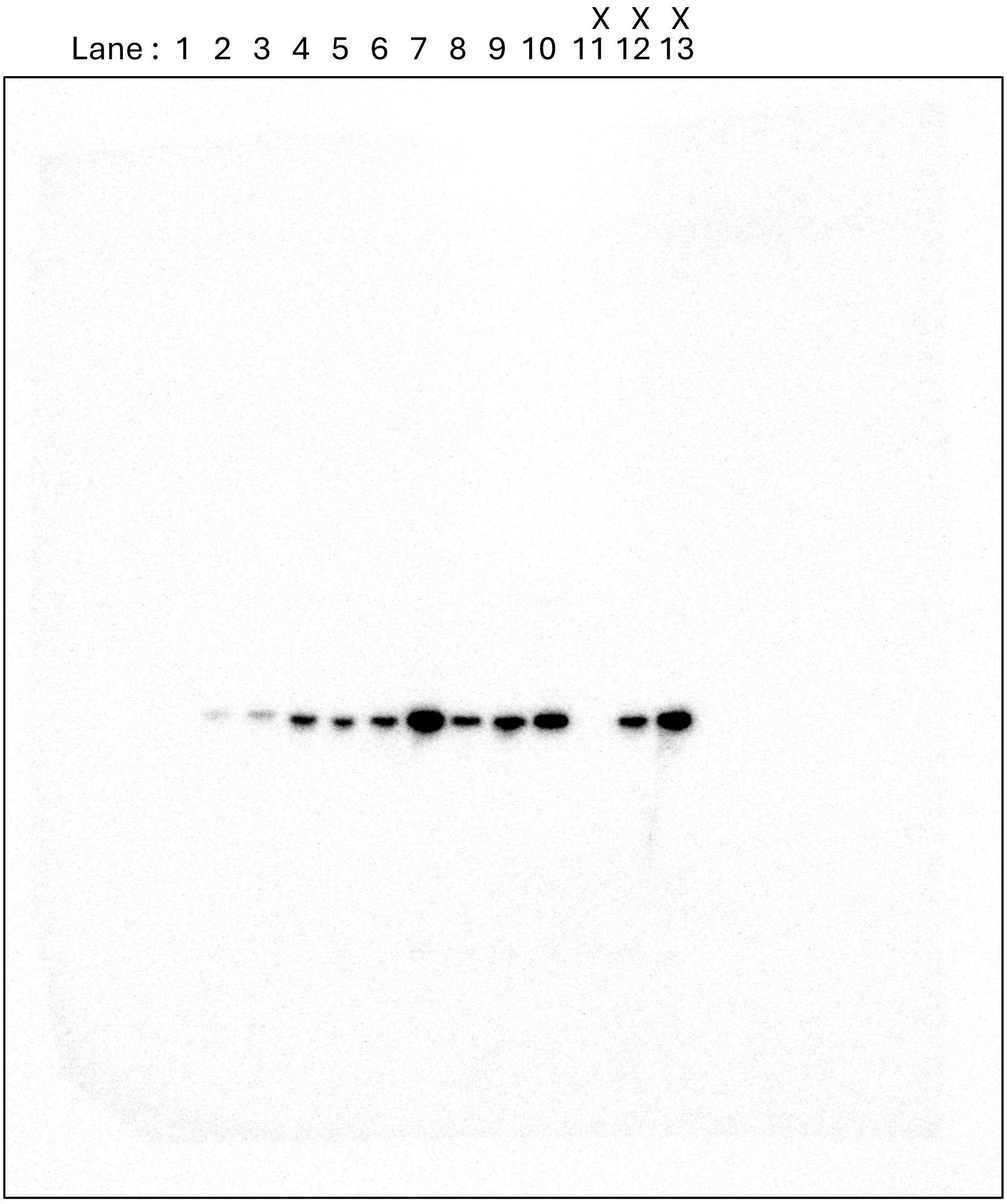

Figure 4C. TBE Urea gel for expression of 5s rRNA in wildtype and HnrS CRISPRi knockdown strains (pCPB vector) in MEM - HEPES

Samples were loaded from left to right as follows:

Lane 1: Ladder (Not Visible in Northern)

Lane 10: EHEC str. Sakai *Δstx1 stx2A::kan* pCPB (wildtype)

Lane 11: EHEC str. Sakai *Δstx1 stx2A::kan* pCPB::sgHnrS (knockdown)

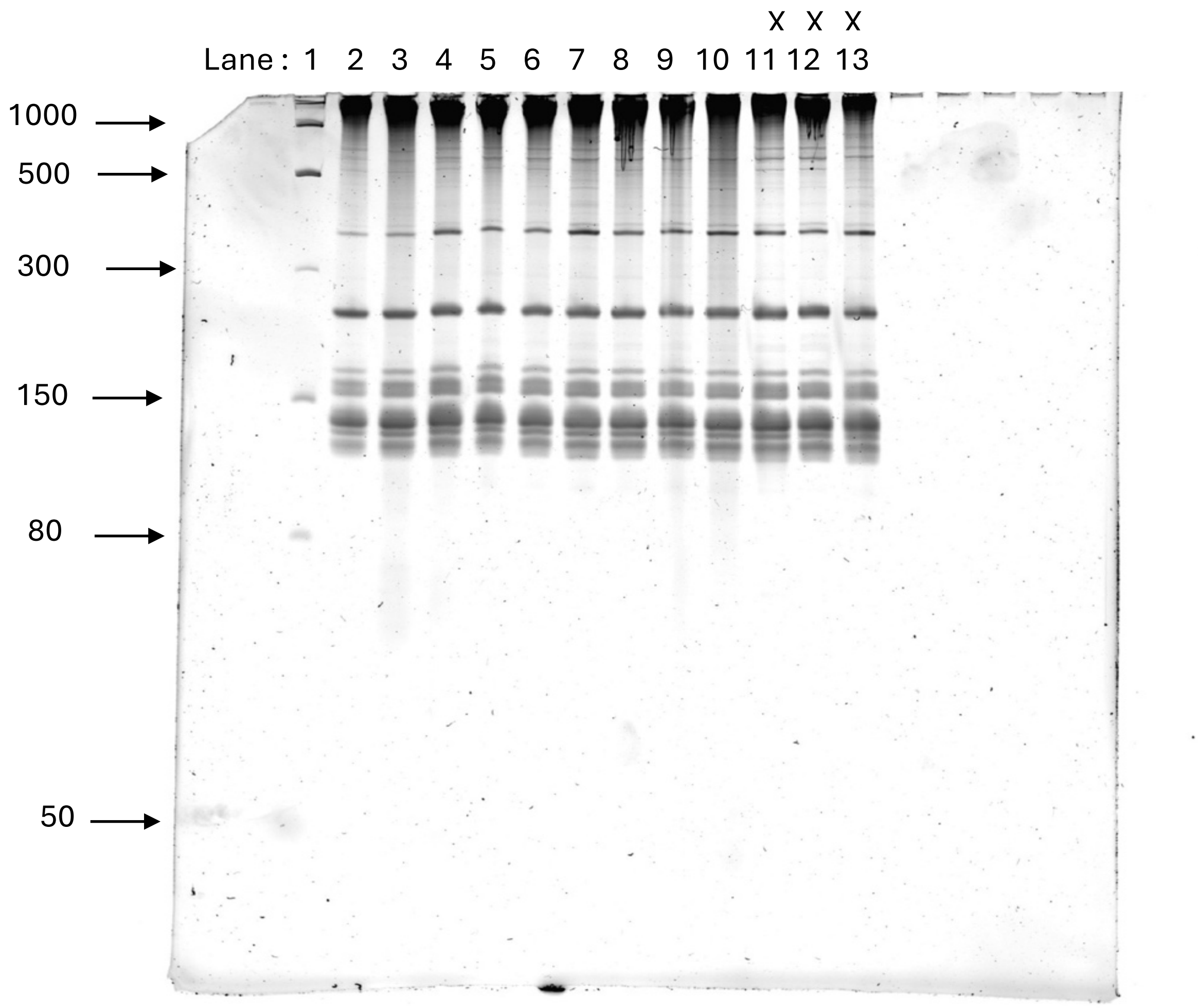

Figure 5A. Coomassie-stained SDS-PAGE gels showing secreted protein profiles. EHEC Sakai stx(-) wild type, wild type with pBR322, *Δhha*, *Δhha* with pBR322, and *Δhha* complemented with pBR322::*hha*

Samples were loaded from left to right as follows:

Lane 1: Ladder
Lane 2: WT
Lane 3: pBR322
Lane 4: *Δhha*
Lane 5: *Δhha* pBR322
Lane 6: *Δhha* pBR322::*hha*

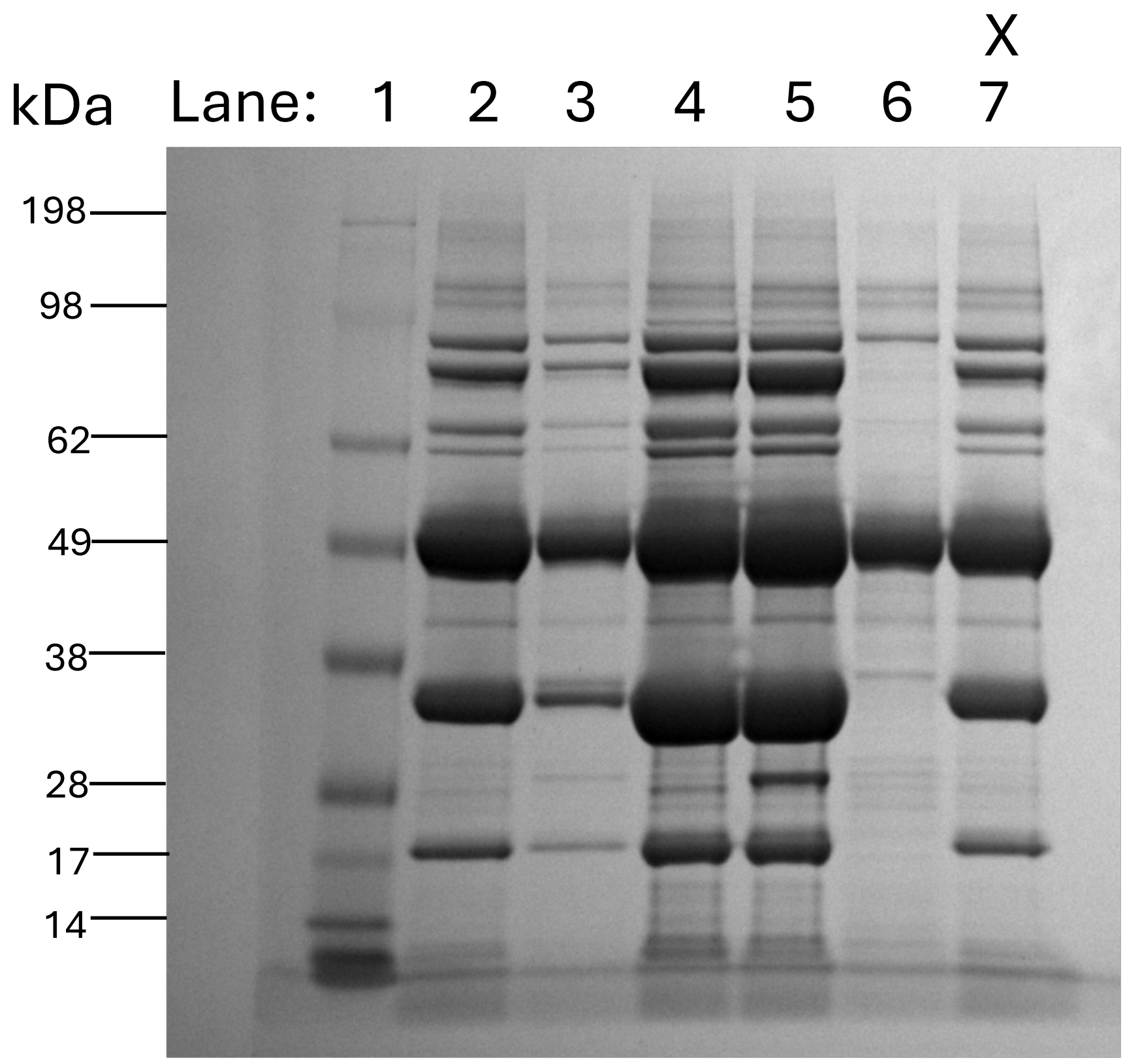

Figure 5B. EHEC TUV 93-0 stx(-) wild type, *Δhha*, and *Δhha* complemented with pBR322::*hha*.

Samples were loaded from left to right as follows:

Lane 1: Ladder

Lane 2: pBR322

Lane 3: *Δhha* pBR322

Lane 4: *Δhha* pBR322::*hha*

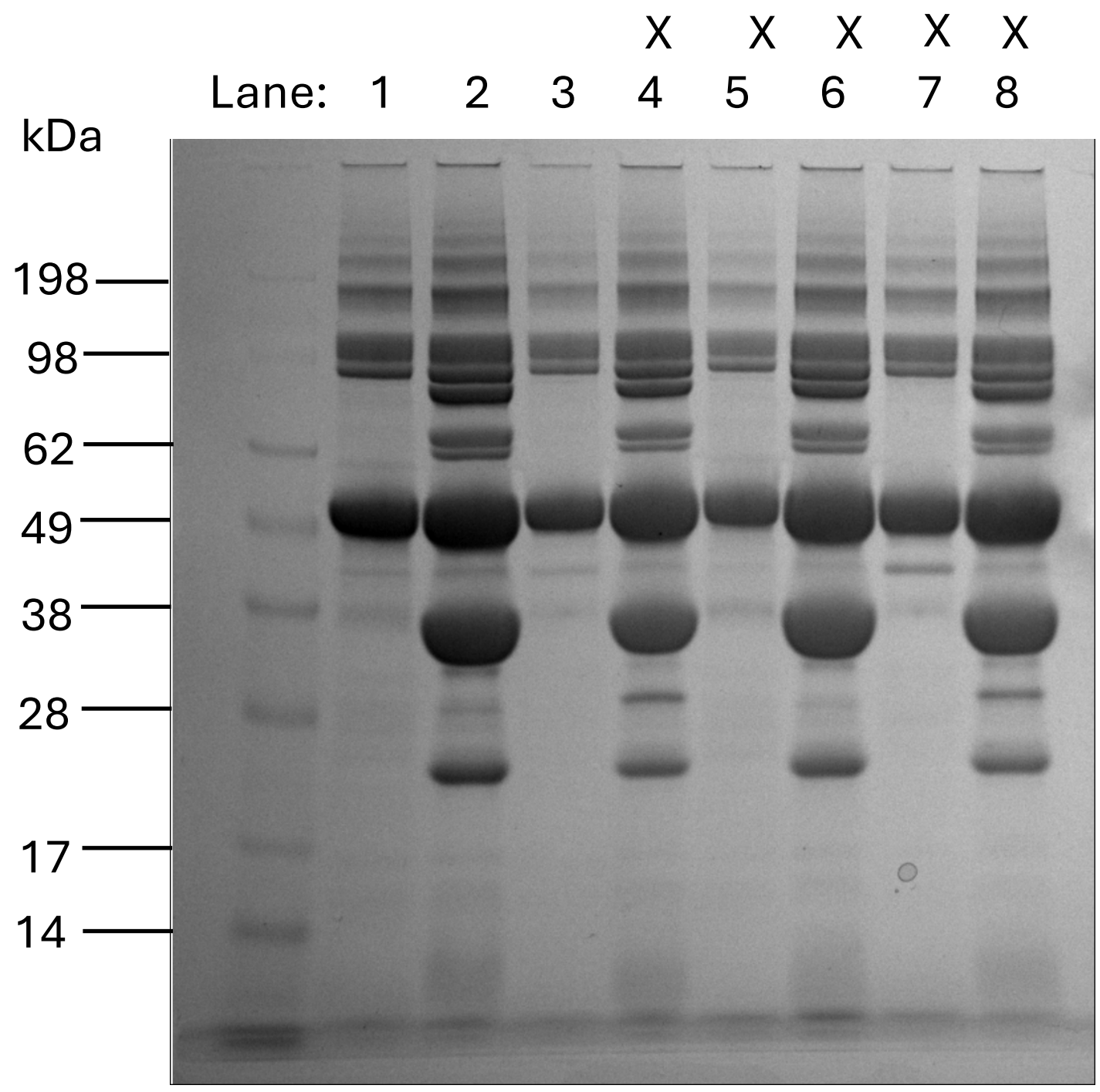

Figure 5C: Western blot analysis of EspD in secreted fractions EHEC str Sakai wild type with pBR322, *Δhha* with pBR322, and *Δhha* complemented with pBR322::*hha*

Samples were loaded from left to right as follows:
Lane 5: pBR322
Lane 6: *Δhha* pBR322
Lane 7: *Δhha* pBR322::*hha*

X X X X X X X
Lane: 1 2 3 4 5 6 7 8 9 10

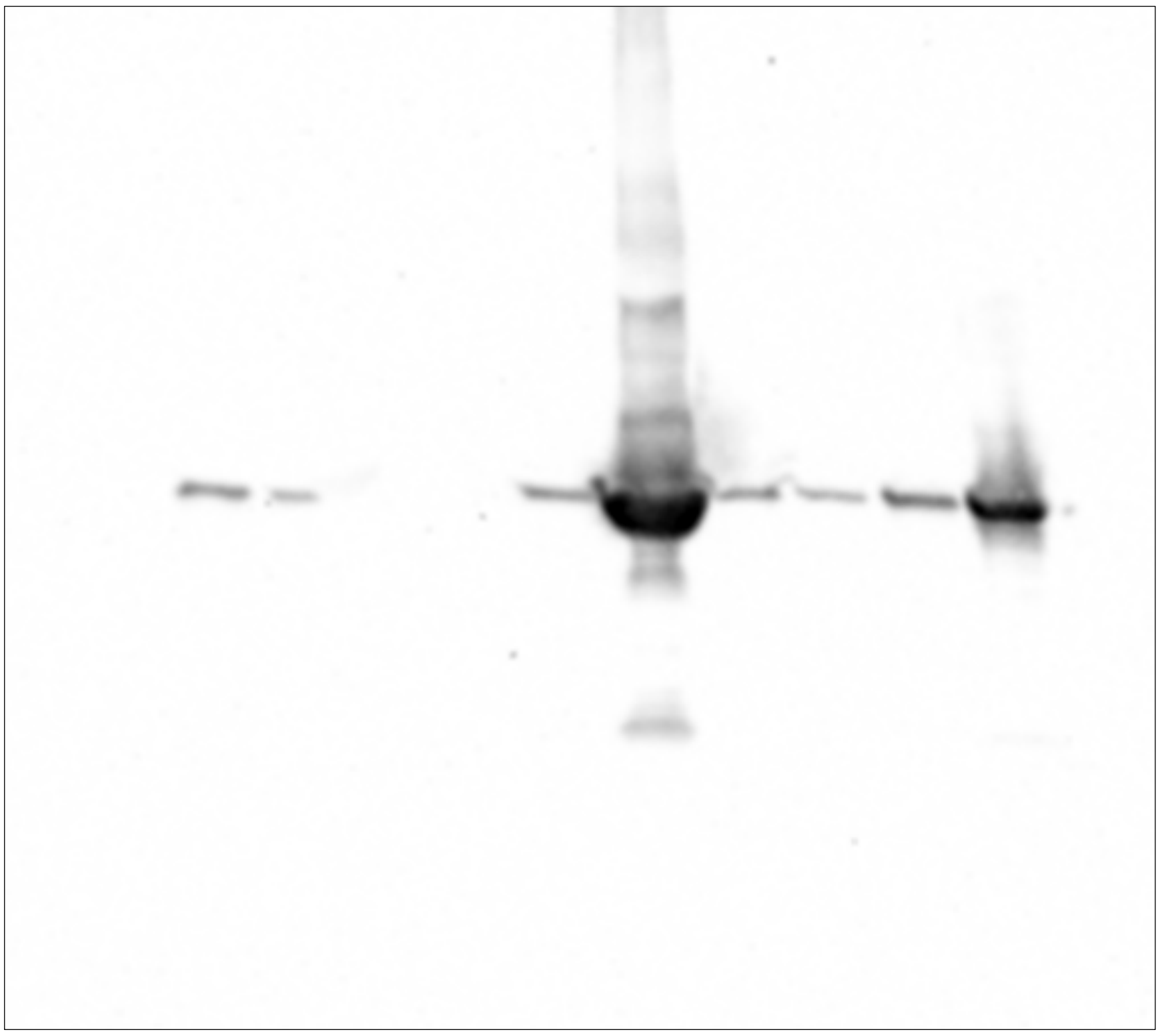

Figure 5C: Western blot analysis of DnaK in whole-cell samples EHEC str Sakai wild type with pBR322, *Δhha* with pBR322, and *Δhha* complemented with pBR322::*hha*

Samples were loaded from left to right as follows:

Lane 5: pBR322
Lane 6: *Δhha* pBR322
Lane 7: *Δhha* pBR322::*hha*

X X X X X X X

Lane: 1 2 3 4 5 6 7 8 9 10

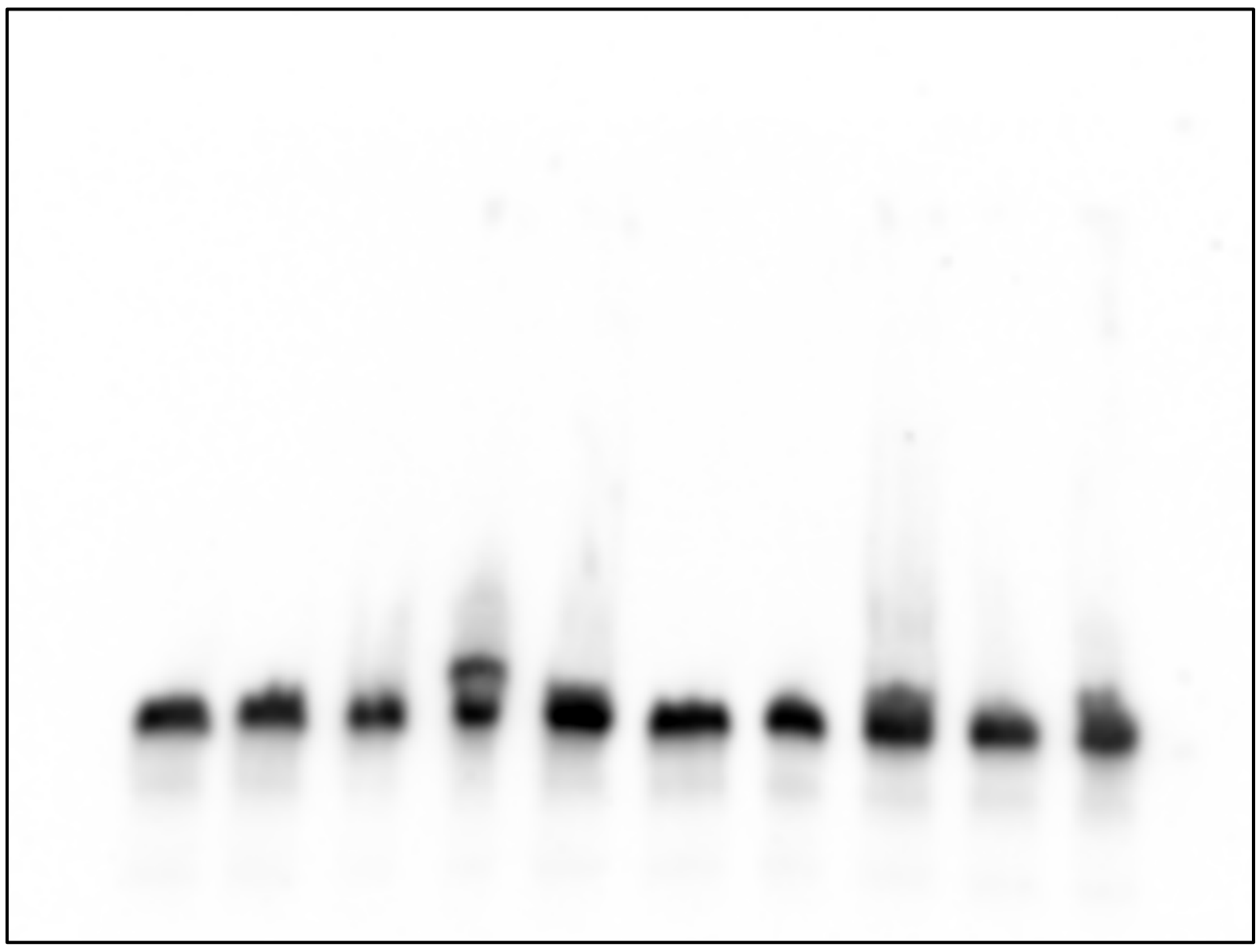

Figure 5D: Western blot analysis of EspD after HnrS knockdown in EHEC Strain backgrounds are indicated above. TUV 93-0 with Empty vector pCPB (pCPB), TUV 93-0 withpCPB::sgHnrS , Sakai with pCPB , and Sakai with pCPB::sgHnrS .

Samples were loaded from left to right as follows:
Lane 1: EHEC str. TUV93-0 pCPB
Lane 2: EHEC str. TUV93-0 pCPB::HnrS sgRNA
Lane 3: EHEC str. Sakai pCPB
Lane 4: EHEC str. Sakai pCPB::HnrS sgRNA

					X	X	X	X	X	X
Lane:	1	2	3	4	5	6	7	8	9	10

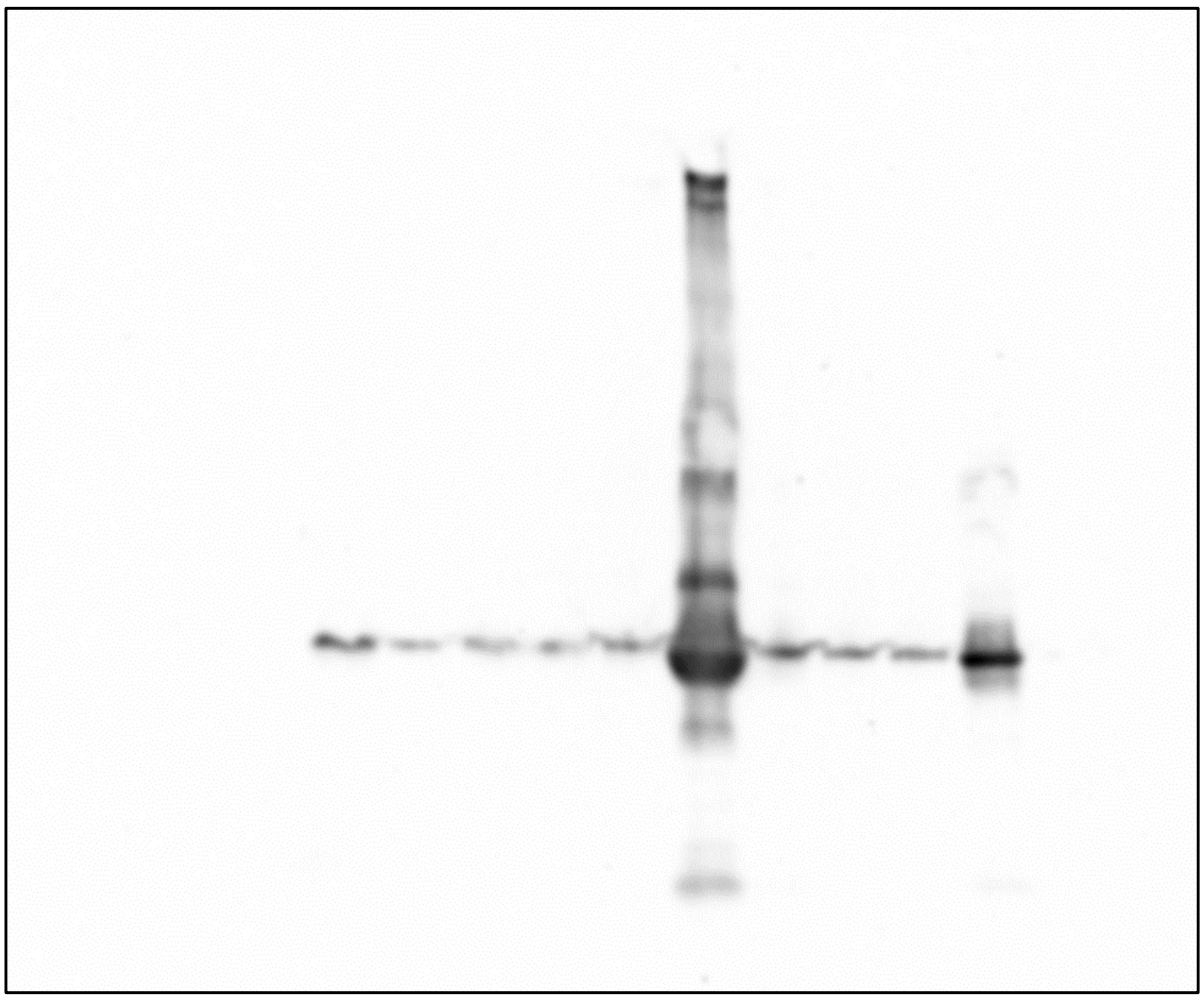

Figure 5D: Western blot analysis of DnaK after HnrS knockdown in EHEC Strain backgrounds are indicated above. TUV 93-0 with Empty vector pCPB (pCPB), TUV 93-0 withpCPB::sgHnrS , Sakai with pCPB , and Sakai with pCPB::sgHnrS .

Samples were loaded from left to right as follows:
Lane 1: EHEC str. TUV93-0 pCPB
Lane 2: EHEC str. TUV93-0 pCPB::HnrS sgRNA
Lane 3: EHEC str. Sakai pCPB
Lane 4: EHEC str. Sakai pCPB::HnrS sgRNA

Lane: 1 2 3 4 5 6 7 8 9 10

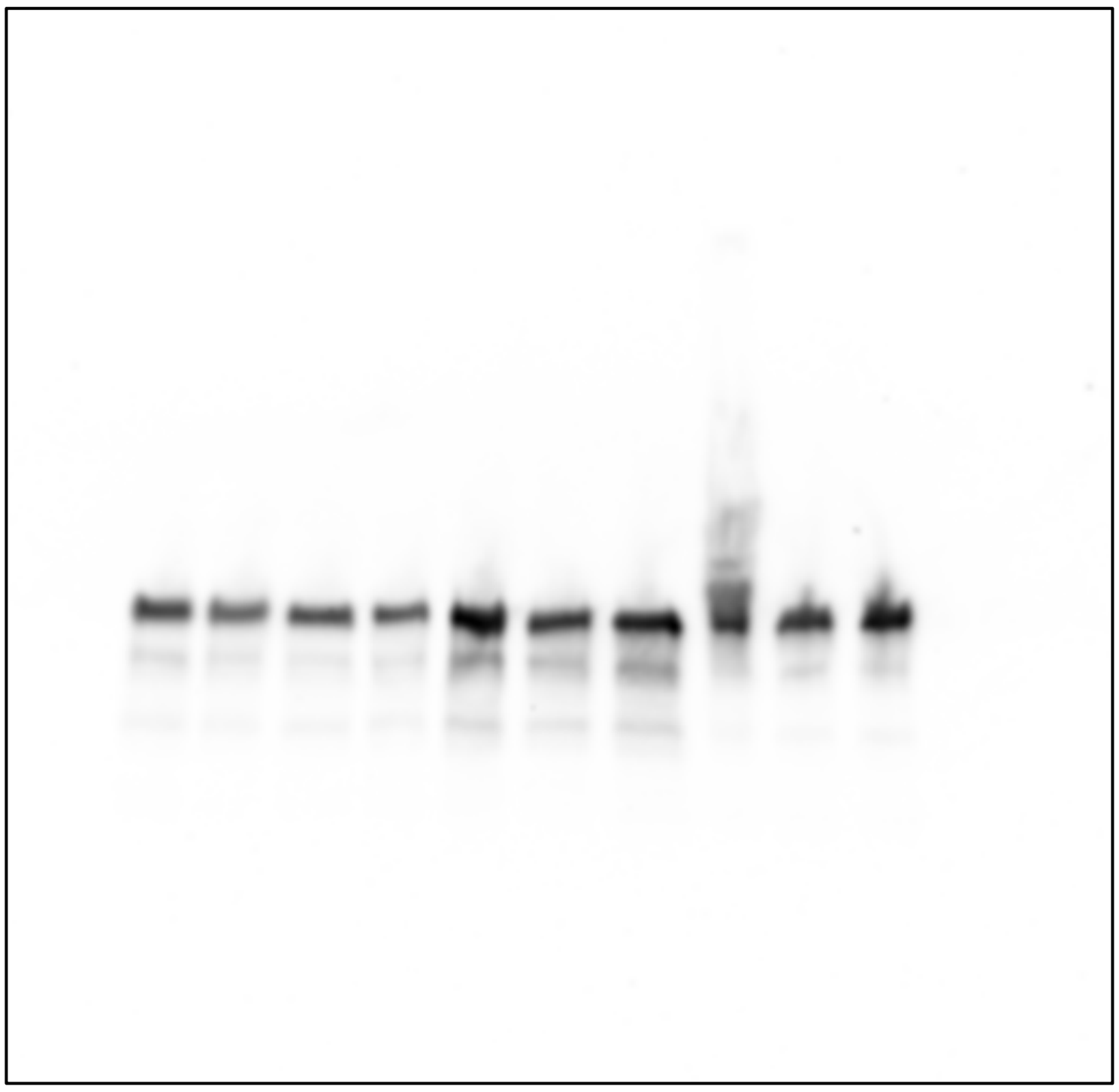

Supplementary Figure 2B and 2C

B&C. EMSA of HnrS interaction with the *mazF* coding sequence *in vitro*. 50fmol of either *mazF* (panel B) or HnrS (panel C) were radiolabelled and incubated with the cognate RNA (concentrations indicated above).
Lanes:

1. 0 nM *mazF'*
2. 10 nM *mazF'*
3. 25 nM *mazF'*
4. 50 nM *mazF'*
5. 100 nM *mazF'*
6. 0 nM HnrS
7. 10 nM HnrS
8. 25 nM HnrS
9. 50 nM HnrS
10. 100 nM HnrS

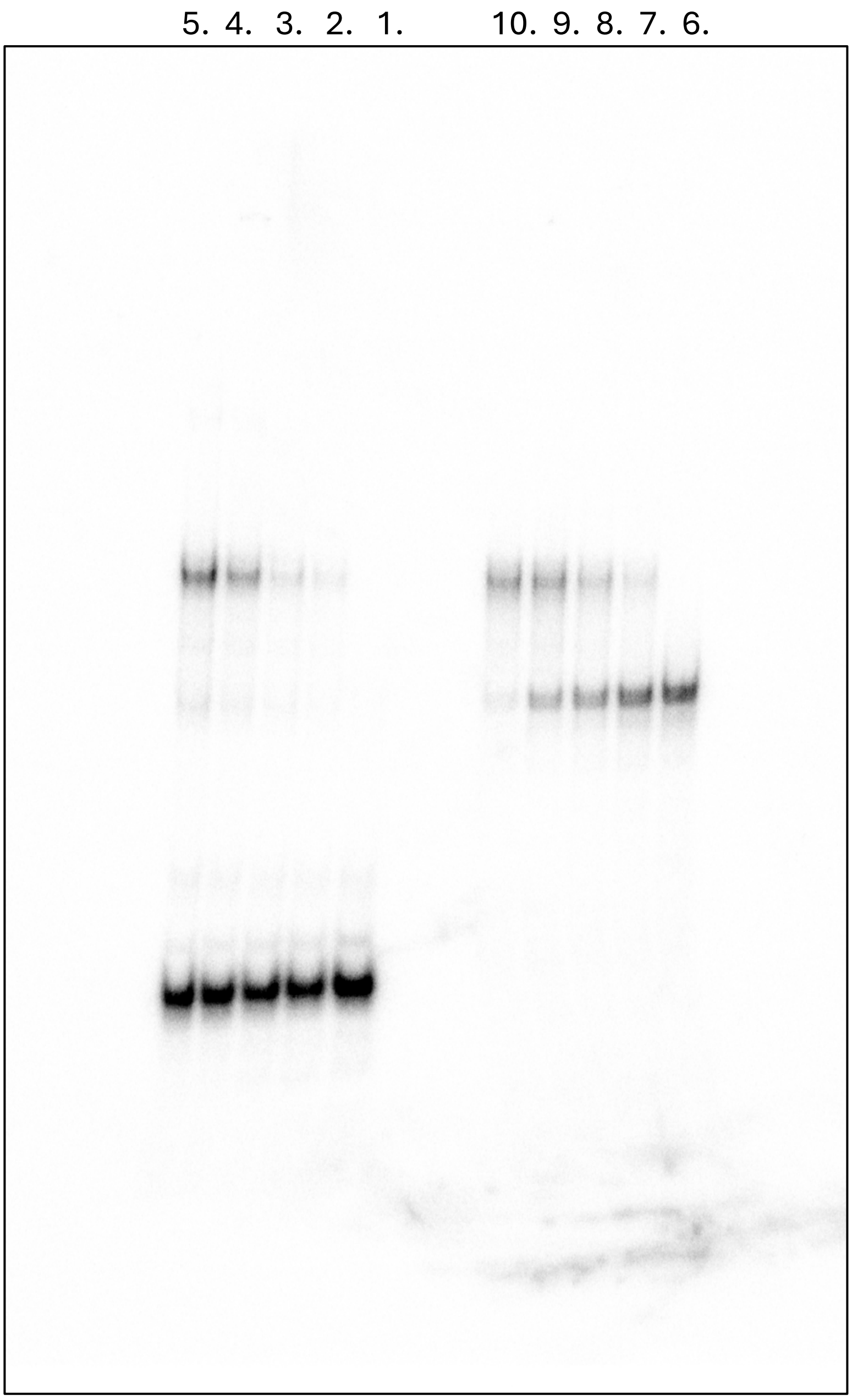

Supplementary Figure 2D : Plaquing assay to assess the sensitivity of E. coli K12 wild-type (left) and ΔmazEFG (right) to a panel of phages, including P1, T4, λ, and V5-like. Serial dilutions of each phage were spot plated onto the respective bacterial strains, and phage plaques were counted to assess bacterial sensitivity.

E. coli str K12 wild-type

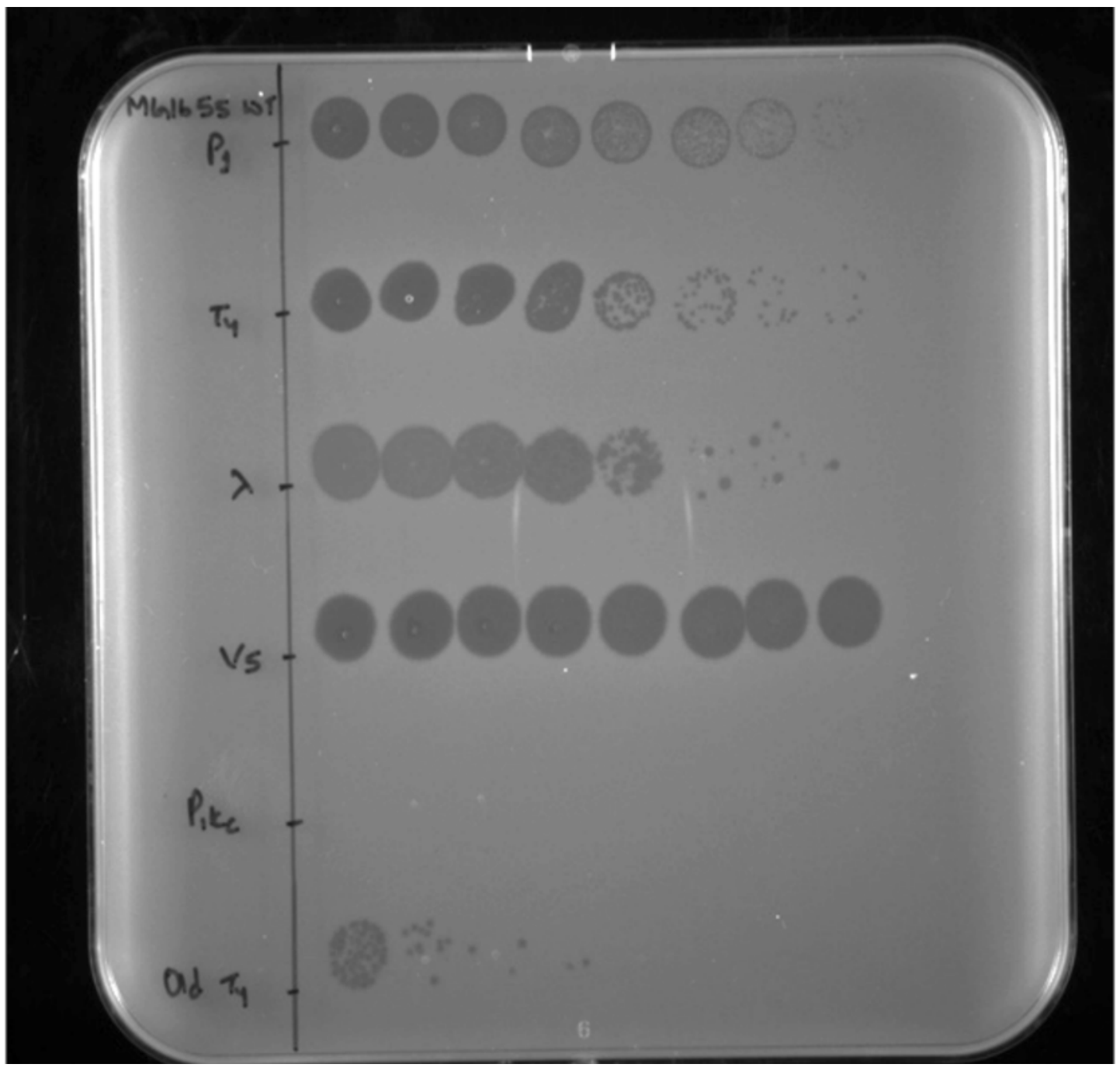

E.coli str K12 Δ *mazEFG*

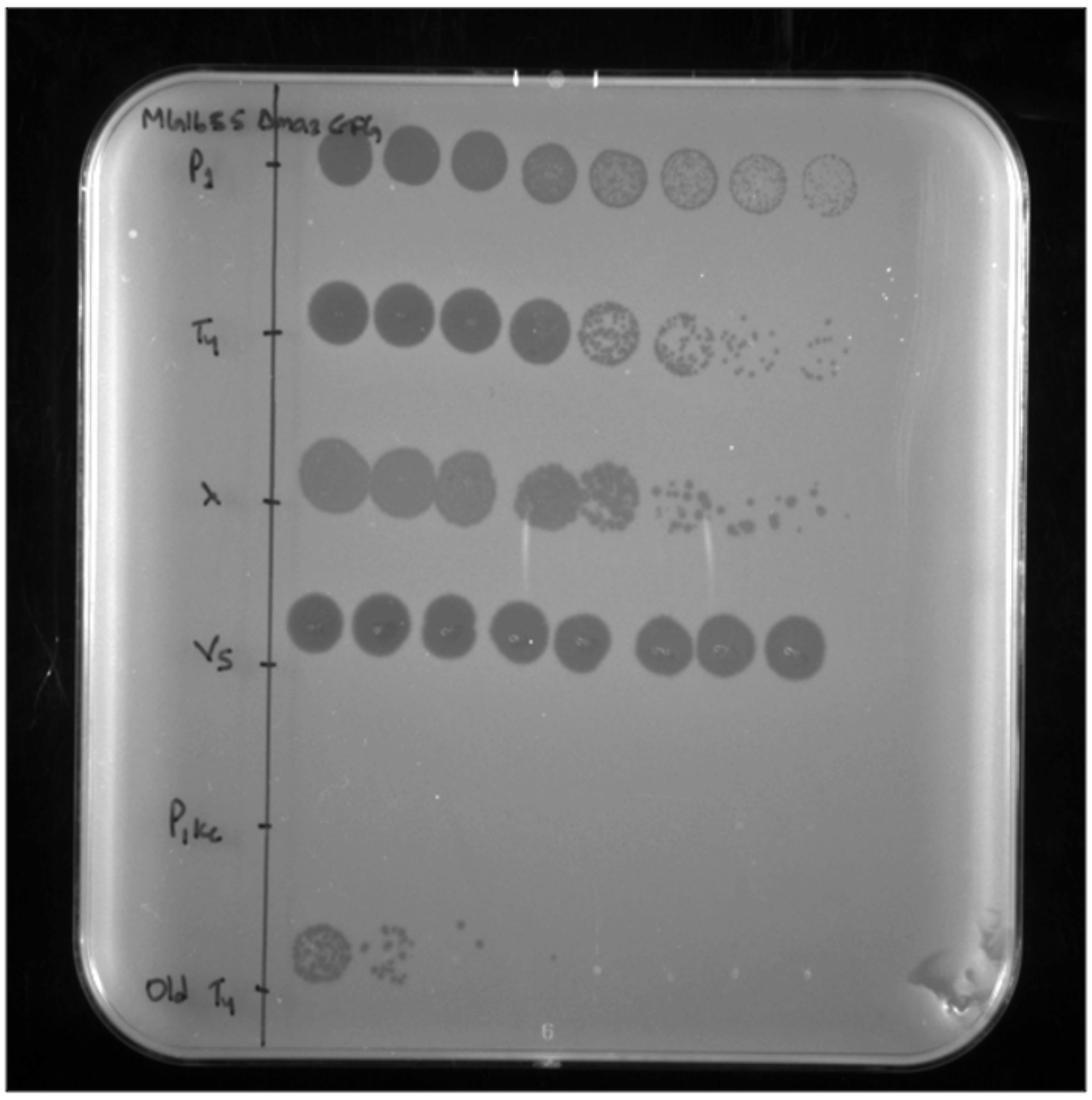

Supplementary Figure 5A. Northern blot showing HnrS expression in wild type (WT), *ΔhnrS*, and complemented strains with pulse expression from pBAD+1::*HnrS*. RNA was harvested at mid-exponential (OD600 = 0.8) and early stationary phase (OD600 = 1.8).

Samples were loaded from left to right as follows:

Lane 1: Ladder (Not Visible in Northern)
Lane 2: Mid-exponential phase (OD600 = 0.8)
Lane 3: Early stationary phase (OD600 = 1.8)

Lanes 4–5: M9 minimal medium
Lane 4: Mid-exponential phase (OD600 = 0.8)
Lane 5: Early stationary phase (OD600 = 1.8)

Lanes 6–7: MEM-HEPES
Lane 6: Mid-exponential phase (OD600 = 0.8)
Lane 7: Early stationary phase (OD600 = 1.8)

X X X X X X X X

Lane : 1 2 3 4 5 6 7 8 9 10 11 12 13 14

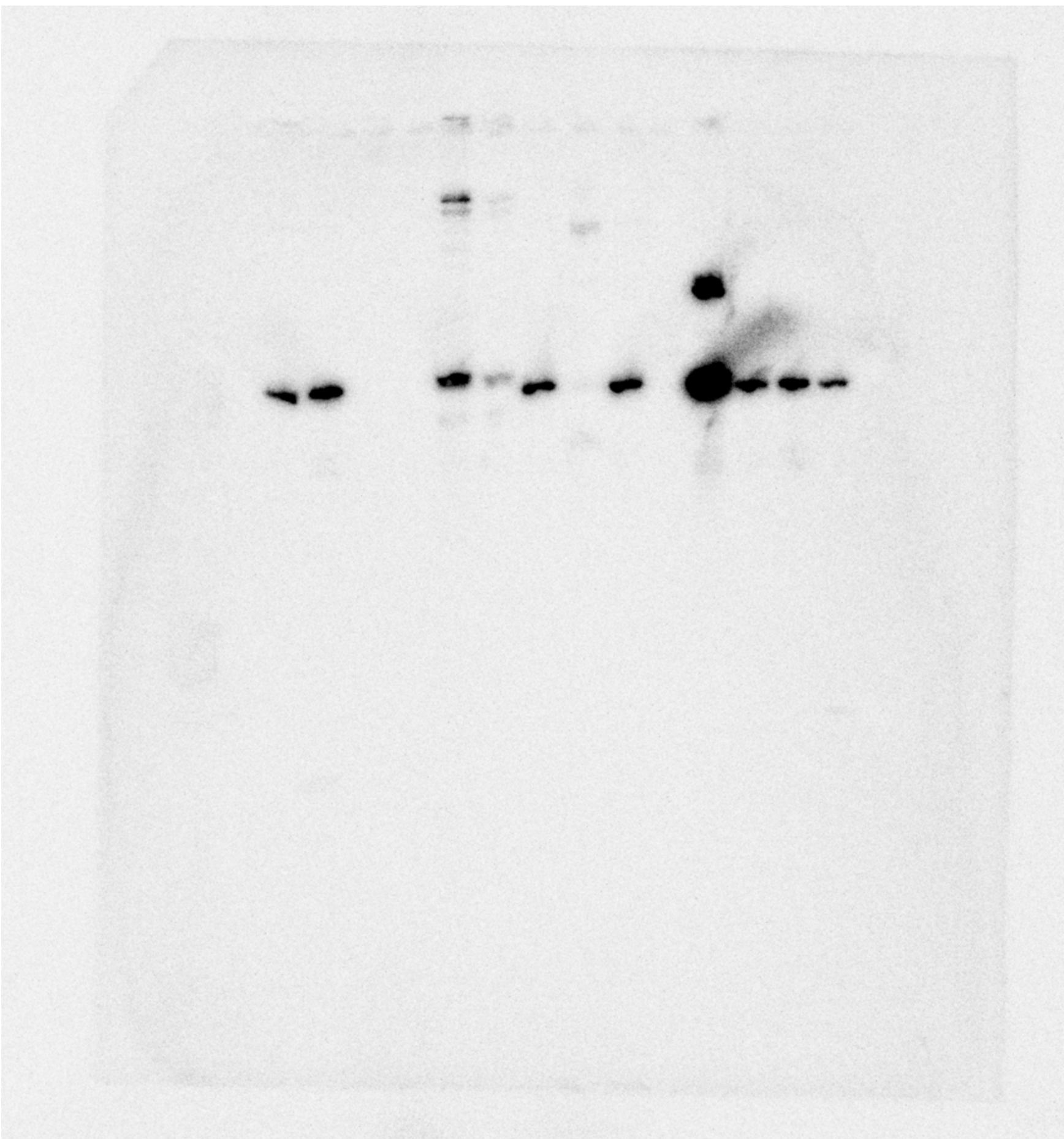

Supplementary Figure 5A. TBE Urea gel for expression of 5s rRNA in wild type (WT), *ΔhnrS*, and complemented strains with pulse expression from pBAD+1::*HnrS*. RNA was harvested at mid-exponential (OD600 = 0.8) and early stationary phase (OD600 = 1.8).

Samples were loaded from left to right as follows:

Lane 1: Ladder (Not Visible in Northern)
Lane 2: Mid-exponential phase (OD600 = 0.8)
Lane 3: Early stationary phase (OD600 = 1.8)

Lanes 4–5: M9 minimal medium
Lane 4: Mid-exponential phase (OD600 = 0.8)
Lane 5: Early stationary phase (OD600 = 1.8)

Lanes 6–7: MEM-HEPES
Lane 6: Mid-exponential phase (OD600 = 0.8)
Lane 7: Early stationary phase (OD600 = 1.8)

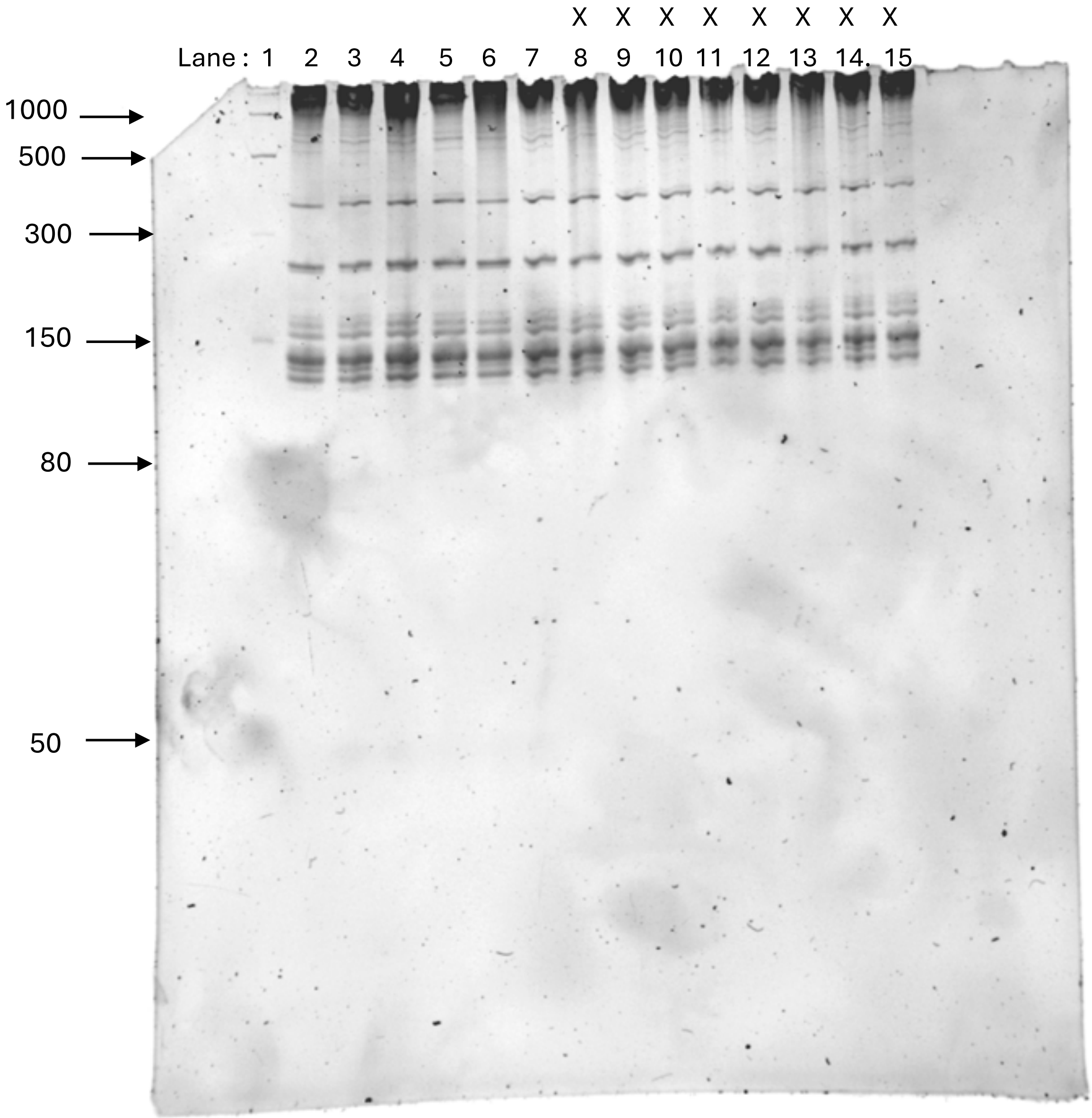

Supplement: Supplementary file 2 [file ppat.1014486.s015.pdf]
